# Dopamine receptors of the rodent fastigial nucleus support skilled reaching for goal-directed action

**DOI:** 10.1007/s00429-023-02685-0

**Published:** 2023-08-24

**Authors:** Violeta-Maria Caragea, Marta Méndez-Couz, Denise Manahan-Vaughan

**Affiliations:** Department of Neurophysiology, Faculty of Medicine, Ruhr-University Bochum, Universitätsstr. 150, MA 4/150, 44780 Bochum, Germany

**Keywords:** Deep cerebellar nuclei, Fastigial nucleus, Dopamine receptors, Skilled reaching, Motor learning, Motivation

## Abstract

The dopaminergic (DA) system regulates both motor function, and learning and memory. The cerebellum supports motor control and the acquisition of procedural memories, including goal-directed behavior, and is subjected to DA control. Its fastigial nucleus (FN) controls and interprets body motion through space. The expression of dopamine receptors has been reported in the deep cerebellar nuclei of mice. However, the presence of dopamine D1-like (D1R) and D2-like (D2R) receptors in the rat FN has not yet been verified. In this study, we first confirmed that DA receptors are expressed in the FN of adult rats and then targeted these receptors to explore to what extent the FN modulates goal-directed behavior. Immunohistochemical assessment revealed expression of both D1R and D2R receptors in the FN, whereby the medial lateral FN exhibited higher receptor expression compared to the other FN subfields. Bilateral treatment of the FN with a D1R antagonist, prior to a goal-directed pellet-reaching task, significantly impaired task acquisition and decreased task engagement. D2R antagonism only reduced late performance post-acquisition. Once task acquisition had occurred, D1R antagonism had no effect on successful reaching, although it significantly decreased reaching speed, task engagement, and promoted errors. Motor coordination and ambulation were, however, unaffected as neither D1R nor D2R antagonism altered rotarod latencies or distance and velocity in an open field. Taken together, these results not only reveal a novel role for the FN in goal-directed skilled reaching, but also show that D1R expressed in FN regulate this process by modulating motivation for action.

**Supplementary Information:**

The online version contains supplementary material available at 10.1007/s00429-023-02685-0.

## Introduction

Dopaminergic signaling, originating in the midbrain, drives information encoding in a multitude of brain regions, including experience encoding in the hippocampus (Lemon and Manahan-Vaughan 2012; Lisman and Grace 2005; Duszkiewicz et al. 2019), reward error prediction in the striatum (Hollerman and Schultz 1998; Cox and Witten 2019), cognitive control in the prefrontal cortex (Jacob et al. 2016; Ott and Nieder 2019), effortful decision-making in the cingulate cortex (Schweimer and Hauber 2006; Cardinal et al. 2001; Assadi et al. 2009), and fear conditioning in the amygdala (Guarraci et al. 1999; Stubbendorff and Stevenson 2021). Although the cerebellum is intrinsically involved in motor control and the optimisation of motor learning, little is known about the role of the dopaminergic system in information processing in this structure (Flace et al. 2021).

In addition to its well-documented roles in coordinating gait and voluntary movement (Flace et al. 2021; Leiner et al. 1994; Schmahmann 1997), the cerebellum has become increasingly investigated as a structure that may contribute to cognitive and emotional processes (Sokolov et al. 2017; Schmahmann 2019; De Zeeuw et al. 2021; Kostadinov and Häusser 2022). Many insights have been gained in this regard from studies of cerebellar disorders in human patients or animal models, where roles for the cerebellum in executive functions and emotional regulation (Schmahmann and Sherman 1998; Manto and Mariën 2015), spatial memory and goal-directed navigation (Babayan et al. 2017; Zhang et al. 2023), verbal working memory (Desmond et al. 2005), social processing (D'Mello and Stoodley 2015), fear conditioning (Koutsikou et al. 2014; Frontera et al. 2020; Vaaga et al. 2020), and reward signaling (Kostadinov and Häusser 2022) have been described. Many of these studies highlight the deep cerebellar nuclei (DCN) as key areas supporting cognitive and affective processes (Schmahmann 2019; Carlson et al. 2021; Sokolov et al. 2017; De Zeeuw et al. 2021; Middleton and Strick 2000; Carta et al. 2019; Pierce and Péron 2020; Adamaszek et al. 2022).

The DCN, that include the fastigial (medial) nucleus (FN), interpositus nucleus (in humans, called the emboliform and globose nuclei, respectively), and dentate (lateral) nucleus, represent the main output of the cerebellum (Voogd 2004). The FN is the most medial and also the smallest and phylogenetically oldest nucleus of the DCN (Zhang et al. 2016). It contributes to axial, proximal, and ocular motor control, and the interpretation of body movement in space (Brooks and Cullen 2013; Shaikh et al. 2005). Recent findings suggest that the FN also participates in affective and autonomic regulation (Zhang et al. 2016) and emotional memory (Adamaszek et al. 2022). Its abovementioned roles in interpreting body movement in space and in supporting spatial navigation raise the question, as to whether the FN may also support goal directed behavior.

In the mammalian brain, DA acts on two classes of receptors: the dopamine D1-like (D1R) receptors (including D1 and D5 receptors), and D2-like (D2R) receptors (including the D2, D3, and D4 receptors) (Beaulieu and Gainetdinov 2011). While DA receptor contribution to motor function has been well studied in terms of the pathogenesis of movement disorders, such as dyskinesia (Cenci 2007), the role of DA receptors in modulating mnemonic processes has mainly been studied from the perspective of their shaping of hippocampal-dependent learning and synaptic plasticity (Hansen and Manahan-Vaughan 2014; Jay 2003). Motor skill learning under dopaminergic modulation has been demonstrated in a multitude of species (Wood 2021), but very few of these studies considered the cerebellar dopaminergic system (Caligiore et al. 2017). Although the cerebellum does not express DA receptors in high amounts (Flace et al. 2021), DA binding (Versteeg et al. 1976; Panagopoulos et al. 1991; Volkow et al. 2013), the DA transporter (DAT), neuronal tyrosine hydroxylase (TH) expression (Delis et al. 2008; Melchitzky and Lewis 2000) and DA receptors (Diaz et al. 1995; Barili et al. 2000; Martres et al. 1985; Mehdizadeh et al. 2020) have all been described in the cerebellum.

The main dopaminergic projections to the cerebellum originate in the ventral tegmental area (VTA) and target the cerebellar cortex and some parts of the DCN (Lazarov et al. 1998; Kim et al. 2009). Other sources of cerebellar DA are the locus coeruleus (Canton-Josh et al. 2022) and TH-positive cerebellar Purkinje cells (Lazarov et al. 1998; Glaser et al. 2006; Kim et al. 2009; Locke et al. 2020). Irrespective of the dopaminergic signal source, D1R and D2R expressed in the DCN have been ascribed roles in nonmotor functions, such as social behavior and cognition (Locke et al. 2018; Heskje et al. 2020; Cutando et al. 2022). Furthermore, monosynaptic efferent projections from the FN to the VTA (Carta et al. 2019), substantia nigra (Snider et al. 1976; Washburn et al.), and locus coeruleus (Snider 1975), have been described. This has led to the hypothesis that a communication loop exists between the cerebellum and dopaminergic nuclei that serves to regulate reward and motivation processes (Herrera-Meza et al. 2014; Hoshi et al. 2005; Wagner et al. 2017; Bostan and Strick 2018; D'Angelo 2019; Carta et al. 2019; Pierce and Péron 2020; Carlson et al. 2021).

The DCN contribution to goal-directed behavior has been proposed to occur via a cortico-cerebellar loop (Gao et al. 2018), or via its connectivity with striatal structures (Contreras-López et al. 2023; Xiao et al. 2018). Other studies point towards a specific role of the FN in goal-directed learning. For example, transient FN perturbation leads to a disruption of subsequent correct responses in a skilled directional licking behavior that was motivated by reward (Gao et al. 2018). The functional role of DA receptors in the FN in learning behavior has not been investigated. In this study, we, therefore, targeted D1R and D2R in the FN using pharmacological antagonists that were locally applied to the FN. We examined to what extent these receptors modulate a skilled reaching task in adult rats. We found that, while D1R antagonism, prior to commencing training, prevents the acquisition of the skilled reaching task and also regulates the level of engagement in goal-directed action, D2R antagonism does not affect these behaviors. D1R antagonism during consolidation had no effects on reaching success but decreased reaching speed and task engagement. Examination of open field and rotarod performance indicated that motor behavior was unaffected by D1R antagonism, but task engagement was affected. Furthermore, we show that D1R antagonism affects motivation for action, but not motor aspects of this task, supporting a role for the FN in cognitive, goal-directed behavior.

## Materials and methods

For all experimental procedures, the guidelines of the European Communities Council Directive of September 22nd, 2010 (2010/63/EU) for the care of laboratory animals were followed. The experiments were approved in advance by the ethics committee of the federal state of North Rhine Westphalia (NRW; Landesamt für Naturschutz, Umweltschultz und Verbraucherschutz, NRW). All efforts were made to keep animal numbers to a minimum.

### Animals

*Long Evans* male rats were housed in ventilated cabinets (Scantainer, Scanbur Technology A/S, Karlslunde, Denmark) where temperature and humidity were kept constant at 22 ± 2 °C and 55 ± 5%, respectively. A 12-h light–dark cycle was followed (lights on at 7 a.m.), with experimental procedures performed during the light cycle. Before starting the experiments, during the open field and rotarod test, food and water were provided ad libitum. For the skilled reaching task, the rats were food-restricted starting with the habituation sessions to reach a body weight that was maximally 10% less than their starting weight.

### Immunohistochemistry

**Fig. 1:**
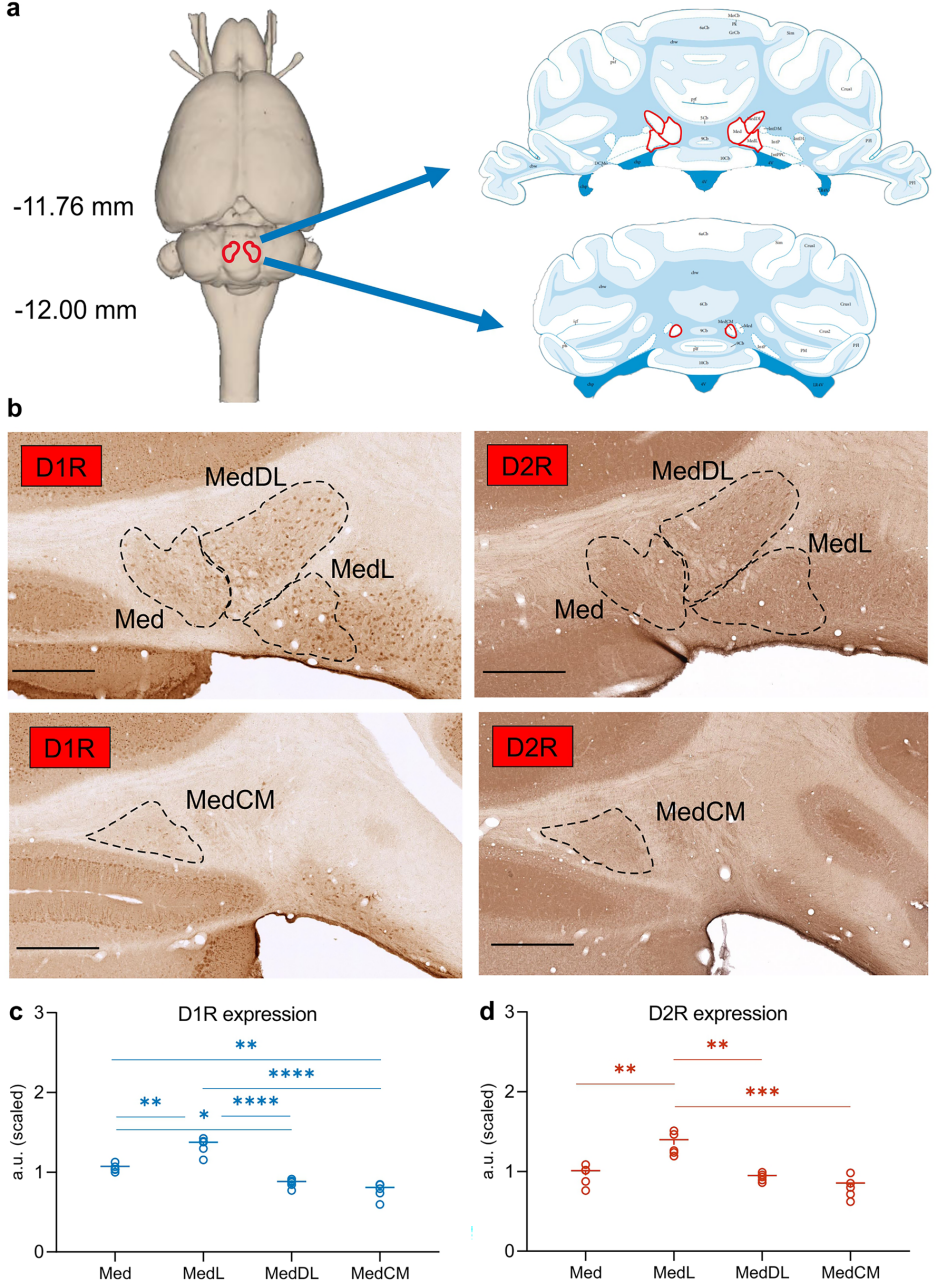
D1R and D2R protein expression in the rat fastigial nucleus (FN). **a** Schematic representation of the rat FN. The left panel indicates the FN position in a 3D horizontal plane (adapted from Paxinos and Watson 2005). The right panel shows coronal sections through the cerebellum: the upper part illustrates a medial view of the deep cerebellar nuclei where three FN subfields can be identified: medial (Med), medial lateral (MedL), and medial dorso-lateral (MedDL). The lower part shows the medial caudo-medial (MedCM) FN subfield (modified illustration from Paxinos and Watson 2005). Areas outlined in red indicate FN and its subfields. **b** Selected regions of interest (ROIs) showing D1R (left) or D2R (right) dopamine receptor immunolabeled cerebellum sections. The upper panels include Med, MedDL and MedL subfields for each receptor, while the lower panels show D1R and D2R protein expression in the MedCM area. The ROIs are labeled by the black dashed lines. Scale bar (in black): 500 µm. **c-d** Quantitative analysis of D1R (**c**) and D2R expression levels (**d**) measured as optical density. t-test, *p < 0.05, **p < 0.01, ***p < 0.001, ****p < 0.0001; *n* = 5. Error bars represent ± SEM

To assess DA receptor expression, animals were euthanized via deep anesthesia. Then, by means of aortal cannula insertion, perfusion with a cooled solution containing 0.2% Heparin mixed with Ringer occurred for 10 min, followed by a 15 min perfusion with 4% paraformaldehyde (PFA). Extracted brains were preserved in PFA for another 24 h at 4 °C and then transferred to a 30% sucrose solution. The cerebellum was sliced in 30 µm-thick coronal serial sections, stored in 96-well plates filled with phosphate buffered saline (PBS) at 4 °C, and one in six sections was stained with cresyl violet (as described below) to select the appropriate slice material for D1R and D2R immunodetection. After choosing the slices that contained the deep cerebellar nuclei for each animal, two sets of three duplicates each were selected—one set including the medial (*Med*), the medial dorsolateral (*MedDL*), and the medial lateral (*MedL*) subfields of the fastigial nucleus (11.3–11.7 mm posterior to bregma), and a second set containing the medial caudomedial (*MedCM*) part (11.8–12.0 mm posterior to bregma)—based on a rat brain atlas (Paxinos and Watson 2005) (Fig. 1a, b). To minimize optical density measurement variations, the immunohistochemistry (IHC) procedures for both receptors were conducted simultaneously for the same animals (two groups of three rat brains for each staining session). Five brains were used for data analysis, as one brain was excluded due to the staining artifacts induced by suboptimal cardiac perfusion.

D1R and D2R IHC experiments were conducted using the avidin–biotin complex (ABC) method (Hsu et al. 1981; Dubovyk and Manahan-Vaughan 2018, 2019). In brief, after three-step washing in Tris-buffered saline (TBS, for D1R), or PBS, (for D2R), we first blocked the endogenous peroxidase by a 20 min pretreatment of the free-floating sections in 0.3% H_2_O_2_. After PBS or TBS rinsing, sections were incubated for 90 min at room temperature (RT) with blocking solution: 0.2% Triton-X in TBS (TBS-Tx) containing 1% bovine serum albumin (BSA) and 20% Avidin, for D1R; or 0.2% Triton-X in PBS (PBS-Tx) containing 10% normal Goat serum (NGS) and 20% Avidin, for DR2. Next, the sections were incubated overnight at RT with primary antibody solutions: either rabbit polyclonal antiD1R (AB1765p, Merck Millipore, Burlington, VT, USA) 1:100 in 0.2% TBS-Tx containing 1% BSA and 20% Biotin; or rabbit polyclonal antiD2R (AB1558, Merck Millipore, Burlington, VT, USA) 1:250 in 0.2% PBS‐Tx containing 1% NGS and 20% Biotin. After three-step TBS or PBS rinsing the next day, the sections were incubated for 90 min with biotinylated goat anti-rabbit (1:500, BA‐1000, Vector Laboratories Burlingame, CA, USA), as the second antibody, in a dilution medium of 1% BSA in 0.1% TBS-Tx, for D1R, and 1% NGS in 0.1% PBS-Tx, for D2R, respectively. After additional three rinsing steps in buffer in both protocols, the sections were incubated for either 30 min (D2R) or 90 min (D1R) at RT with an ABC kit (PK‐6100, Vector Laboratories, Burlingame, CA, USA), 1:1,000 in the same dilution medium as for the second antibody step. For the D1R protocol, an additional amplification step (Adams 1992) was performed where the brain sections were incubated with biotinylated tyramide (bT) in a solution of 10 µl bT + 10 µl 0.01% H_2_O_2_ in 1,000 µl TBS. After 20 min, the D1R-immunolabeled sections were washed in TBS (4 × 2 min) and another ABC kit incubation was performed for 30 min followed by one TBS + two PBS 10 min rinsing rounds. As the final step for each protocol, after rinsing, the slices were treated with 3,3′-diaminobenzidin (DAB, Sigma-Aldrich, Darmstadt, Germany, 5 mg) and 0.01% H_2_O_2_ in 10 ml PBS medium for approx. 10 min. After three more PBS rinsing steps, the sections were mounted on gelatine-coated adherent glass slides (ThermoFisher Scientific, Waltham, MA, USA) and air-dried overnight. Next day, the slices were dehydrated in ascending concentrations of alcohol, cleared with xylene, and coverslipped with dibutylphthalate polystyrene xylene (DPX Mountant for histology, Sigma-Aldrich, Darmstadt, Germany) for further microscopic analyses. Negative control tests, where the receptor antibodies were omitted from the protocols described above, were performed to exclude confounds (not shown). The effectiveness of the D1R and D2R antibodies used here was previously confirmed using Western blotting (Dubovyk and Manahan-Vaughan 2019; Zou et al. 2005).

### Surgery

All animals included in the behavioral tests underwent stereotaxic chronic implantation of bilateral guide cannulas in the FN at 7–8 weeks of age (coordinates: − 11.5 mm anterior from bregma; ± 1.1 mm from midline; approx. − 5 mm ventral from skull). This was done under sodium pentobarbital anesthesia (52 mg/kg), administered intraperitoneally. The implanted cannulas were manufactured from stainless steel hypodermic needles (25 G in diameter, 0.5 × 16 mm RW/LB, Henry Schein Inc., New York, NY, USA), adjusted to a length of 1.4 cm. Two stainless steel fixation screws were inserted in the skull bone anterior from bregma. Both the screws and the cannulas were sealed with surgical glue and, then, dental acrylic was added to build a stable socket.

### Ligand delivery

The dopamine D2R antagonist (S)-(–)-3-bromo-N-[(1-ethyl-2-pyrrolidinyl) methyl]-2,6 dimethoxybenzamide (remoxipride; Tocris, Bristol, UK) and the D1R antagonist (R)-( +)-7-Chloro-8-hydroxy-3-methyl-1-phenyl-2,3,4,5-tetrahydro-1H-3-benzazepine hydrochloride (SCH23390 or SCH, Tocris, Bristol, UK) were dissolved in physiological saline solution (0.9% NaCl) to obtain a dose of 10 µg/µl for remoxipride and of 5.94 µg/µl for SCH, respectively. The doses were selected based on previous findings in freely behaving rats, where hippocampal synaptic plasticity was inhibited by these ligand doses, without affecting basal synaptic transmission (Manahan-Vaughan and Kulla 2003; Hagena and Manahan-Vaughan 2016; Caragea and Manahan-Vaughan 2021).

One microliter of the ligand-containing solution, or vehicle (for control experiments), was microinjected in each FN over a period of four minutes, at the speed of 0.25 µl/min, followed by an additional minute of waiting time before the injection cannula was removed from the guide cannula (Shimizu et al. 2014). The injection was delivered via a precision injector (Hamilton syringe, Reno, NV, USA) that was connected by tubing to a 33 G diameter needle (0.26 × 13 mm, Luminject, Transcodent GmbH, Kiel, Germany), that was 1.5 cm in length. All injections were carried out 30 min prior to the behavioral manipulations.

### Postmortem verification of cannula positions

Following the conclusion of the behavioral experiments, animals were euthanised by means of deep anesthesia. The cannulas were filled with methylene blue (MB) solution (1% MB in Saline, Sigma-Aldrich, Darmstadt, Germany) to mark each cannula tip in the DCN. Brains were extracted and stored in 4% paraformaldehyde (PFA) solution (phosphate buffered saline, 0.025 M of PFA, pH of 7.4) at 4 °C. One week later, they were transferred to a 30% sucrose solution for cryoprotection. Three to five days later, the tissue was sliced into 30 µm-thick frozen slices using a freezing microtome (Cuttec S sliding microtome, SLEE Medical GmbH, Nieder-Olm, Country). A digital camera was used to take pictures of the frozen cerebellum slices whenever MB staining was perceived. The slices were collected in 96-well plates filled with PBS. Sections of the region of interest were mounted on adherent glass slides. After air-drying, the slices were stained in 0.1% cresyl violet (Hansen and Manahan-Vaughan 2015). Each section was examined under a light microscope (Leica Mikrosysteme Vertrieb GmbH, Wetzlar, Germany) to determine the terminal MB labeling and gliosis signifying the position of the cannula tips. The relevant slices were scanned using a high precision slide scanner (Axio Scan.Z1, Zeiss, Jena, Germany) for more fine-grained analysis (ZEN 2 software, Blue edition, Zeiss, Jena, Germany) and documentation of the location of cannula tips. The data sets from animals with misplaced cannulas were excluded from further analysis. The reconstruction of cannula tip placement for all included animals, is shown in Fig. 3a.

### Behavioral experiments

In order to avoid interference of task-related learning effects, the animals participated in each of the three behavioral tasks in a randomized order. For each rat, handling was performed on 5 consecutive days (minimum 5 min daily) before starting the behavioral procedures. Tasks were conducted at a minimum of 7 days apart from one another.

#### Skilled reaching task

The task was adapted from the skilled forelimb reaching task developed for rats (Whishaw and Pellis 1990). In summary, the animals were trained over multiple days to reach for food by approaching and retrieving single pellets with their forelimb through a narrow pellet access slit. For this, we used an apparatus, built in-house, that was inspired by the work of Zemmar (2015). It consisted of an operant reaching chamber (35 cm L × 14 cm W × 33 cm H), with a one-cm wide vertical slit in the middle of the front wall. Four centimeters from the floor, a platform (9 cm L × 4 cm W) was attached to the outside of the front wall to allow for pellet display. The platform had three small indentations engraved 1.5 cm in front of the pellet access slit—one corresponding to the center of the pellet access slit and two others located at 1.5 cm distance left and right, to guide pellet placement. The distances were selected so as to prevent the retrieval of pellets using the tongue. The rear wall was made of gray opaque acrylic while the lateral and front walls, as well as the platform, were made of transparent plastic. The upper side of the platform was covered with a black non-reflective sticker to facilitate video scoring. One HD video camera (30 frames per second) was installed 20 cm above the platform, covering a surface ca. 15 cm × 15 cm wide, to allow scoring both the time the animal spent near the pellet access slit and the reaching attempts through it. For a sample of the initial experimental cohorts, another side camera was installed to screen for potential motoric side effects of the drugs. The average light intensity at the platform level was ca. 70 lx (dim light). The food pellets used (Dustless Precision Pellets™, 45 mg, Rodent Purified Diet, Bio-Serv Inc, Flemington, NJ, USA) were of uniform size and shape, and small enough for the animal to easily grasp and retract.

The skilled reaching task (SRT) started with five pre-training days: In the first three days, food restriction was implemented, and the animals explored the SRT box daily for 10 min. A handful of pellets were distributed on the floor, and on the front platform, very close to the pellet access slit. On the next two days, for 15 min daily, the rats learned to approach the front of the apparatus and reach for the pellets placed on the outside platform, right in the opening of the pellet access slit. Once the rat took the pellet, another pellet was dropped into the back of the box (at the wall opposite the pellet access slit), so as to shape the behavior of the animal, and to encourage the animal to leave the slit location in order to go to the back of the apparatus and then subsequently return to the slit. This was needed to circumvent excessively frequent reaching attempts (once the pellet was removed) later on during the training trials (Metz et al. 2005; Zemmar 2015).

For the next five days, the animals were trained to reach for pellets with a single forepaw through the pellet access slit. The first day session included up to 20 initial trials conducted to determine the preferred reaching forepaw. During these trials, the single pellets were placed in the center indentation on the platform. When an animal made more than 7 out of 10 attempts with the same forepaw, this was designated the preferred paw, and subsequently, pellets were placed on the indentation contralateral to this paw. In cases where a clear paw preference was not established, ten further trials were conducted. If even then, the animal still did not show a paw preference, the pellets were placed in the center indentation for all of the following training trials. The first training day session lasted for 20 min, while the following four daily sessions lasted for 15 min. This training period was called the *acquisition* phase. Following the conclusion of acquisition, daily testing sessions were conducted for three days (*early test, ET*), comprising 20 trials/day in which a pellet should be withdrawn, each of which lasted no longer than 15 min. Seven days later, long-term memory of the acquired skill was tested on three more consecutive days (*late test, LT*). After each session for an individual animal, the walls of the chamber were thoroughly cleaned with 70% ethanol, washed with water, and dried.

#### Rotarod

Rats were trained in a rotarod (RR) treadmill (ROTAROD for RAT 47750, UgoBasile srl, Gemonio, Italy) to test for grip strength, balance, or motor coordination alterations induced by D1R and D2R antagonists. The task was conducted on three consecutive days, with a daily session consisting of three trials separated by approx. 30 min intervals. The first day consisted of a habituation session, where the animals needed to remain balanced on the rod that turned at the rate of 5 rotations per minute (rpm) for a maximum of 300 s. On the second and third days, representing the actual test, the rotarod accelerated at an increasing speed from 4 to 40 rpm for up to 300 s. Thirty minutes before the start of the first trial of the second day, vehicle, or ligand, was bilaterally injected in the FN, while on the third day no injection occurred. Irrespective of the session, a trial ended when the rat fell off the rod, or when the 300 s had elapsed. The latency to fall was recorded for each individual animal.

#### Open field test

A dark gray opaque acrylic arena (76 cm L × 76 cm W × 55 cm H) served as the apparatus for the test. The bottom of the field was covered with black and gray patterned washable plastic, selected for suitable discrimination of the rat by the video tracking system. The arena was surrounded by an opaque curtain and the experimental area was faintly but uniformly illuminated (approx. 80 lx). The animal behavior was recorded by means of a monitoring system, live tracking, and subsequent analysis with EthoVision XT software (v14, Noldus Information Technology BV, Wageningen, The Netherlands) and Solomon Coder software (version: beta 19.08.02, https://solomoncoder.com). Prior to the testing session, the animals were injected with vehicle, or a DA receptor antagonist, as described above, and then transferred to the experimental room for 30 min of waiting time. No prior habituation to the arena was conducted before the actual testing, to avoid a decrease in exploration behavior during the test. At the beginning of the open field test (OFT), the rats were placed in the arena facing the centre point of the north wall and were allowed to freely explore the open field for 5 min. The floor was thoroughly cleaned with ethanol 70%, washed with water and dried, after each testing trial.

#### Elevated plus maze

A plus sign-shaped maze made of dark gray opaque acrylic was used for this task. The maze has two open and two closed arms (each of 12 cm wide and 60 cm long from center) and was elevated at a distance of 50 cm from the floor. The junction of the four arms formed a 15 by 15 cm area. The animals were first injected with vehicle or ligand-containing solution, as described above, and 30 min later the elevated plus maze (EPM) test started, with each individual rat being placed in the center area of the maze. Each animal was allowed to explore the maze for 5 min, while being live video-tracked (Ethovision, Noldus Information Technology BV, Wageningen, The Netherlands).

### Data analysis

For the *optical density analysis* of DA receptor expression, we used a similar approach as before (Dubovyk and Manahan-Vaughan 2019). In brief, we initially selected the slices of interest by means of light microscope inspection and subsequently scanned them with a high-precision slide scanner (Axio Scan.Z1, Zeiss, Jena, Germany). The scanned sections in the field of view were further cropped to contain the regions of interest that were defined based on a rat brain atlas (Paxinos and Watson 2005) as described above. For each brain, one slice was selected for each FN subfield (either right or left hemisphere) as shown in Fig. 1b. To control for labeling intensity discrepancies, the measurements within each slice were normalized to the same region comprising the cerebellar white matter (*cbw*), i.e., the portion between lobules 5/6 and lobule 9, that characteristically is devoid of neurotransmitter receptor expression (Barili et al. 2000) (Fig. 1a**)**. The images containing the delineated areas of interest were analyzed with the FIJI/ImageJ software (National Institute of Health, Bethesda, MD, USA) to calculate receptor density using a custom-made code. Deconvolution of color information was used to convert images into 8-bit format and increase the dynamic range of the signal. We then subtracted background staining values (the *cbw* selection) to obtain the raw values of the optical density measurement for each slice. Finally, to scale the resulting values obtained from two independent staining sessions, we used an R software (RStudio) algorithm using a generalized residual sum of the squares as a scaling strategy (Kemmer et al. 2022).

In the *skilled reaching task*, all acquisition and test sessions were scored offline with the Solomon Coder software (version: beta 19.08.02, https://solomoncoder.com), during which the operator was blind to the animal’s treatment. Based on an approach described by others (Nica et al. 2017), animal behavior was manually scored for each video-recorded session for the following aspects: *pellet* (every time a pellet was made available for the animal to reach), *reach* (when the rat stretched its forepaw through the pellet access slit in the direction of an available pellet), *miss* (when the animal did not touch the pellet after a reach), *grasp* (when the animal touched the pellet with its forepaw fingers), *slip* (when the rat touched, but failed to retract the pellet), *retract* (successful grasping of a pellet and passing it through the slit in the cage), *drop* (when the pellet was passed through the slit, but it fell down before being put in the mouth), *success* (when the pellet was reached, grasped, retracted and put into the mouth without errors). Furthermore, the time spent within a 5 cm distance to the pellet access slit (nose oriented towards the slit wall), from the total time of a session, was measured with the same software. For comparisons within and between groups, the following values were computed for each session for individual animals: *success ratio* (number of successes divided by the number of pellets, multiplied by 100); *pellet reaching speed* (number of pellets divided by the total time of a session); *error improvement rates for slips* (number of grasps minus the number of slips, divided by number of pellets): *error improvement rates* for *drops* (number of retractions minus number of drops, divided by the number of pellets); *time spent near the slit* (calculated as a percentage of the total session time). No rate was calculated for the *misses* due to the super unitary relationship between reaching attempts and pellets.

For *rotarod* analysi*s*, the *latency to fall*, calculated as the time that elapsed from the timepoint of placing the animal on the rod until its fall, was analyzed for each rat in every habituation or testing trial. In a few cases, where a rat experienced more than one trial where it turned and jumped from the rod (usually, within less than 10 s), all datasets of that animal were excluded. This was the case for two animals. To compare variations within the same animal between treatment and non-treatment sessions, the trials were averaged for each session.

In the *open field test*, prior to experimental exploration recordings, a map of the arena was plotted (EthoVision software, Noldus, Wageningen, The Netherlands). The field was divided into three virtual square-shaped areas (see inset of Fig. 6c): *center* (40 × 40 cm central square), *middle* (56 × 56 cm, 8 cm width area surrounding the center), and *borders* (10 cm wide area bordering the walls of the field). An experimenter, who was unaware of the treatment of individual animals, independently analyzed velocity, distance traveled in the open field, the time spent in each of the areas of the field, and the center area crossing frequency as recorded by means of video-tracking (Ethovision, Noldus Information Technology BV, Wageningen, The Netherlands). Counts of fecal boli, rearing, and grooming bouts, were measured with the Solomon Coder software.

For the *elevated plus maze test*, the total time spent in the open or closed arms was separately calculated (measured in seconds, from the total 300 s duration of the task). For that, a map of the maze was plotted beforehand using EthoVision software (Noldus Information Technology BV, Wageningen, The Netherlands), where the open and closed arms and the center area were outlined to allow the automatic scoring of the time an animal spent in each area during a test session.

All raw data (optical density of receptor expression, exploration values and other behavioral counts in the open field, rotarod latencies, and video scored values for the skilled reaching behavior), were statistically analyzed, and visualized using Prism 9 software (GraphPad Software Inc., Boston, MA, USA). Corel Draw (Corel Corporation, Ottawa, Canada; version 20.0.0.633, 2018) was used to compose the final figures. All data sets passed a normality test (Kolmogorov–Smirnov test of normal distribution). To examine statistical differences of receptor expression across the FN subfields, we used one-way repeated measures analysis of variance (rmANOVA), whereby the areas of interest comprised a repeated-measures factor. Tukey’s post-hoc test was used to correct for multiple comparisons of paired subfields. For analysis of differences within and between-treatment groups for the skilled reaching task, we used a similar approach one-way rmANOVA for within-group changes, and two-way rmANOVA for comparing between-treatment groups, with treatment and session as factors. Furthermore, a Fisher’s LSD test, or paired Student’s t-tests, were used to detect differences between individual time points. Standard deviation calculation was used to compare between means of late test performance of the groups with D1R antagonist delivered prior to acquisition or early test. We used two-way rmANOVA to compare rotarod data between treatment groups, with treatment and individual trial as factors. Paired Student’s t-tests were performed to compare between means of treatment and non-treatment trials within each treatment group. Finally, for the open field test, the ambulation values (total distance traveled and velocity) for each treatment group were compared with controls using a one-way ANOVA accompanied by a Dunnett test to correct for multiple comparisons. Here, the time spent by the animals in each delineated area of the arena was compared between groups using multiple unpaired Student’s t-tests, corrected with the Holm-Sídák test. To compare numbers of fecal boli and the counts of rearing and grooming bouts, unpaired t-tests were used to compare between the scores of pairs of treatment groups for each type of event. For all tests and tasks, significant differences were reported as *p < 0.05, **p < 0.01, ***p < 0.001, or ****p < 0.0001, with values expressed as mean values ± the standard error of the mean (SEM). A summary of all statistical results is shown in Table 1.

**Table 1:** Summary of Statistical results

a.			
D1R	One-way rmANOVA: F_3,12_ = 33.69; ****p < 0.0001		*n* = 5
	Med vs. MedL (Tukey)	**p = 0.0034	
	Med vs. MedDL (Tukey)	*p = 0.0331	
	Med vs. MedCM (Tukey)	**p = 0.0022	
	MedL vs. MedDL (Tukey)	****p < 0.0001	
	MedL vs. MedCM (Tukey)	****p < 0.0001	
D2R	One-way rmANOVA: F_3,12_ = 14.03; ***p = 0.0003		*n* = 5
	Med vs. MedL (Tukey)	**p = 0.0044	
	MedL vs. MedDL (Tukey)	**p = 0.0027	
	MedL vs. MedCM (Tukey)	***p = 0.0002	

b.					
Vehicle Acquisition	One-way ANOVA, F_10,60_ = 4.400, ***p = 0.0001				*n* = 7
	T(1) vs ET(6) (Fisher’s LSD)		**p = 0097		
	T(1) vs ET(7) (Fisher’s LSD)		*p = 0.0281		
	T(1) vs ET(8) (Fisher’s LSD)		**p = 0.0031		
	T(1) vs LT(9) (Fisher’s LSD)		***p = 0.0005		
	T(1) vs LT(10) (Fisher’s LSD)		****p < 0.0001		
	T(1) vs LT(11) (Fisher’s LSD)		***p = 0.0003		
D1R Acquisition	One-way ANOVA, F_10,60_ = 1.370, ^ns^*p* = 0.2164				*n* = 7
D2R Acquisition	One-way ANOVA, F_10,60_ = 3.199, **p = 0.0024				*n* = 7
	T(1) vs ET(6) (Fisher’s LSD)		**p = 0052		
	T(1) vs ET(7) (Fisher’s LSD)		****p < 0.0001		
	T(1) vs ET(8) (Fisher’s LSD)		***p = 0008		
	T(1) vs LT(9) (Fisher’s LSD)		**p = 0.0052		
	T(1) vs LT(10) (Fisher’s LSD)		**p = 0.0015		
	T(1) vs LT(11) (Fisher’s LSD)		***p = 0.0002		
Between treatments	Veh vs D1R	Two-way rmANOVA, Treatment F_1, 12_ = 7.465, *p = 0.0182; Session F_10, 120_ = 4.805, ****p < 0.0001; Session x Treatment: F_10, 120_ = 1.116, ^ns^*p* = 0.3558			*n* = 7
		T(4) (Fisher’s LSD)		*p = 0.0363	
		T(5) (Fisher’s LSD)		^#^*p* = 0.0754	
		ET(6) (Fisher’s LSD)		*p = 0.0159	
		ET(7) (Fisher’s LSD)		*p = 0.0285	
		ET(8) (Fisher’s LSD)		*p = 0.0141	
		LT(9) (Fisher’s LSD)		**p = 0.0040	
		LT(10) (Fisher’s LSD)		**p = 0.0010	
		LT(11) (Fisher’s LSD)		*p = 0.0211	
	Veh vs D2R	Two-way rmANOVA, Treatment F_1, 12_ = 3.088, ^ns^*p* = 0.1043; Session F_10, 120_ = 5.741, ****p < 0.0001; Session x Treatment: F_10, 120_ = 1.800, ^#^*p* = 0.0677			
		LT(9) (Fisher’s LSD)		*p = 0.0156	
		LT(10) (Fisher’s LSD)		**p = 0.0071	
	D1R vs D2R	Two-way rmANOVA, Treatment F_1, 12_ = 0.0646, ^ns^*p* = 0.8037; Session F_10, 120_ = 3.119, **p = 0.0014; Session x Treatment: F_10, 120_ = 1.414, ^ns^*p* = 0.1819			
D1R Test	One-way ANOVA (acquisition), F_4,24_ = 9.445, ****p < 0.0001				*n* = 7
	T(1) vs T(2) (Fisher’s LSD)			*p = 0128	
	T(1) vs T(3) (Fisher’s LSD)			***p = 0.001	
	T(1) vs T(4) (Fisher’s LSD)			***p = 0.001	
	T(1) vs T(5) (Fisher’s LSD)			****p < 0.0001	
	One-way ANOVA (test sessions), F_5,30_ = 1.653, ^ns^*p* = 0.1765				
	T(5) vs ET(6) (paired)			*t*_6_ = 2.102, ^#^p = 0.0803	
	T(5) vs ET(7) (paired)			*t*_6_ = 1.353, ^ns^*p* = 0.2250	
	T(5) vs ET(8) (paired)			*t*_6_ = 2.234, ^#^*p* = 0.0669	

c.			
Vehicle Acquisition	One-way ANOVA, F_10,60_ = 12.51, ****p < 0.0001		*n* = 7
	T(1) vs ET(6) (Fisher’s LSD)	****p < 0.0001	
	T(1) vs ET(7) (Fisher’s LSD)	****p < 0.0001	
	T(1) vs ET(8) (Fisher’s LSD)	****p < 0.0001	
	T(1) vs LT(9) (Fisher’s LSD)	****p < 0.0001	
	T(1) vs LT(10) (Fisher’s LSD)	****p < 0.0001	
	T(1) vs LT(11) (Fisher’s LSD)	****p < 0.0001	
D1R Acquisition	One-way ANOVA, F_10,60_ = 8.439, ****p < 0.0001		*n* = 7
	T(1) vs ET(6) (Fisher’s LSD)	*p = 0.0323	
	T(1) vs ET(7) (Fisher’s LSD)	***p = 0.0007	
	T(1) vs ET(8) (Fisher’s LSD)	*p = 0.0163	
	T(1) vs LT(9) (Fisher’s LSD)	*p = 0.0206	
	T(1) vs LT(10) (Fisher’s LSD)	***p = 0.0005	
	T(1) vs LT(11) (Fisher’s LSD)	****p < 0.0001	
D2R Acquisition	One-way ANOVA, F_10,60_ = 5.466, ****p < 0.0001		*n* = 7
	T(1) vs ET(6) (Fisher’s LSD)	*p = 0.0321	
	T(1) vs ET(7) (Fisher’s LSD)	**p = 0.0034	
	T(1) vs ET(8) (Fisher’s LSD)	**p = 0.0040	
	T(1) vs LT(9) (Fisher’s LSD)	**p = 0.0014	
	T(1) vs LT(10) (Fisher’s LSD)	****p < 0.0001	
	T(1) vs LT(11) (Fisher’s LSD)	****p < 0.0001	
Between treatments	Veh vs D1R	Two-way rmANOVA, Treatment F_1, 12_ = 0.9100, ^ns^*p* = 0.3589; Session F_10, 120_ = 19.40, ****p < 0.0001; Session x Treatment: F_10, 120_ = 1.076, ^ns^*p* = 0.3856	*n* = 7
	Veh vs D2R	Two-way rmANOVA, Treatment F_1, 12_ = 0.5042, ^ns^*p* = 0.4912; Session F_10, 120_ = 16.33, ****p < 0.0001; Session x Treatment: F_10, 120_ = 0.6392, ^ns^*p* = 0.7777	
	D1R vs D2R	Two-way rmANOVA, Treatment F_1, 12_ = 0.018, ^ns^*p* = 0.8968; Session F_10, 120_ = 13.50, ****p < 0.0001; Session x Treatment: F_10, 120_ = 0.5966, ^ns^*p* = 0.8141	
D1R Test	One-way ANOVA (acquisition), F_4,24_ = 6.556, **p = 0.0010		*n* = 7
	T(1) vs T(3) (Fisher’s LSD)	*p = 0.0384	
	T(1) vs T(4) (Fisher’s LSD)	*p = 0.0142	
	T(1) vs T(5) (Fisher’s LSD)	****p < 0.0001	
	One-way ANOVA (test sessions), F_5,30_ = 25.24, ****p < 0.0001		
	ET(6) vs LT(9) (Fisher’s LSD)	**p = 0.0038	
	ET(6) vs LT(10/11) (Fisher’s LSD)	****p < 0.0001	
	ET(7) vs LT(9–11) (Fisher’s LSD)	****p < 0.0001	
	ET(8) vs LT(9) (Fisher’s LSD)	***p = 0.0006	
	ET(8) vs LT(10/11) (Fisher’s LSD)	****p < 0.0001	
	T(5) vs ET(6) (paired)	*t*_6_ = 2.550, *p = 0.0435	
	T(5) vs ET(7) (paired)	*t*_6_ = 3.787, **p = 0.0091	
	T(5) vs ET(8) (paired)	*t*_6_ = 2.263, ^#^*p* = 0.0643	
	T(5) vs LT(9) (paired)	*t*_6_ = 1.383, ^ns^*p* = 0.2160	
	T(5) vs LT(10) (paired)	*t*_6_ = 9.313, ****p < 0.0001	
	T(5) vs LT(11) (paired)	*t*_6_ = 6.384, ***p = 0.0007	

d.					
Vehicle Acquisition	One-way ANOVA, F_10,60_ = 4.052, ***p = 0.0003				*n* = 7
	T(1) vs ET(6) (Fisher’s LSD)		**p = 0047		
	T(1) vs ET(7) (Fisher’s LSD)		**p = 0.0063		
	T(1) vs ET(8) (Fisher’s LSD)		***p = 0006		
	T(1) vs LT(9) (Fisher’s LSD)		**p = 0.0011		
	T(1) vs LT(10) (Fisher’s LSD)		****p < 0.0001		
	T(1) vs LT(11) (Fisher’s LSD)		****p < 0.0001		
D1R Acquisition	One-way ANOVA, F_10,60_ = 1.288, ^ns^*p* = 0.2578				*n* = 7
D2R Acquisition	One-way ANOVA, F_10,60_ = 3.537, **p = 0.0010				*n* = 7
	T(1) vs ET(6) (Fisher’s LSD)		**p = 0064		
	T(1) vs ET(7) (Fisher’s LSD)		**p = 0064		
	T(1) vs ET(8) (Fisher’s LSD)		*p = 0.0120		
	T(1) vs LT(9) (Fisher’s LSD)		**p = 0.0037		
	T(1) vs LT(10) (Fisher’s LSD)		**p = 0.0014		
	T(1) vs LT(11) (Fisher’s LSD)		****p < 0.0001		
Between treatments	Veh vs D1R	Two-way rmANOVA, Treatment F_1, 12_ = 2.852, ^ns^*p* = 0.1171; Session F_10, 120_ = 3.775, ***p = 0.0002; Session x Treatment: F_10, 120_ = 1.048, ^ns^*p* = 0.4084			*n* = 7
		T(4) (Fisher’s LSD)		*p = 0.0267	
		ET(7) (Fisher’s LSD)		^#^*p* = 0.0937	
		ET(8) (Fisher’s LSD)		^#^*p* = 0.0861	
		LT(10) (Fisher’s LSD)		*p = 0.0378	
	Veh vs D2R	Two-way rmANOVA, Treatment F_1, 12_ = 1.699, ^ns^*p* = 0.2169; Session F_10, 120_ = 6.642, ****p < 0.0001; Session x Treatment: F_10, 120_ = 1.112, ^ns^*p* = 0.3585			
	D1R vs D2R	Two-way rmANOVA, Treatment F_1, 12_ = 0.0020, ^ns^*p* = 0.9648; Session F_10, 120_ = 2.674, **p = 0.0055; Session x Treatment: F_10, 120_ = 1.075, ^ns^*p* = 0.3867			
D1R Test	One-way ANOVA (acquisition), F_4,24_ = 7.895, ***p = 0.0003				*n* = 7
	T(1) vs T(3) (Fisher’s LSD)			***p = 0.004	
	T(1) vs T(4) (Fisher’s LSD)			**p = 0.013	
	T(1) vs T(5) (Fisher’s LSD)			****p < 0.0001	
	One-way ANOVA (test sessions), F_5,30_ = 1.393, ^ns^*p* = 0.2550				
	T(5) vs ET(6) (paired)			*t*_6_ = 2.473, *p = 0.0483	
	T(5) vs ET(7) (paired)			*t*_6_ = 1.590, ^ns^*p* = 0.1629	
	T(5) vs ET(8) (paired)			*t*_6_ = 2.367, ^#^*p* = 0.0558	

e.				
Vehicle Acquisition	One-way ANOVA, F_10,60_ = 5.483, ****p < 0.0001			*n* = 7
	T(1) vs ET(6) (Fisher’s LSD)		***p = 0.0008	
	T(1) vs ET(7) (Fisher’s LSD)		**p = 0.0023	
	T(1) vs ET(8) (Fisher’s LSD)		***p = 0.0001	
	T(1) vs LT(9) (Fisher’s LSD)		****p < 0.0001	
	T(1) vs LT(10) (Fisher’s LSD)		****p < 0.0001	
	T(1) vs LT(11) (Fisher’s LSD)		****p < 0.0001	
D1R Acquisition	One-way ANOVA, F_10,60_ = 1.354, ^ns^*p* = 0. 2238			*n* = 7
D2R Acquisition	One-way ANOVA, F_10,60_ = 3.434, **p = 0.0013			*n* = 7
	T(1) vs ET(6) (Fisher’s LSD)		**p = 0.0049	
	T(1) vs ET(7) (Fisher’s LSD)		****p < 0.0001	
	T(1) vs ET(8) (Fisher’s LSD)		***p = 0.0007	
	T(1) vs LT(9) (Fisher’s LSD)		**p = 0.0060	
	T(1) vs LT(10) (Fisher’s LSD)		***p = 0.0007	
	T(1) vs LT(11) (Fisher’s LSD)		***p = 0.0001	
Between treatments	Veh vs D1R	Two-way rmANOVA, Treatment F_1, 12_ = 4.520, ^#^*p* = 0.0549; Session F_10, 120_ = 5.514, ****p < 0.0001; Session x Treatment: F_10, 120_ = 1.412, ^ns^*p* = 0.1830		*n* = 7
		LT(9) (Fisher’s LSD)	*p = 0.0194	
		LT(10) (Fisher’s LSD)	**p = 0.0054	
		LT(11) (Fisher’s LSD)	*p = 0.0454	
	Veh vs D2R	Two-way rmANOVA, Treatment F_1, 12_ = 2.107, ^ns^*p* = 0.1723; Session F_10, 120_ = 7.874, ****p < 0.0001; Session x Treatment: F_10, 120_ = 1.603, ^ns^*p* = 0.1136		
		LT(9) (Fisher’s LSD)	*p = 0.0417	
		LT(10) (Fisher’s LSD)	*p = 0.0232	
	D1R vs D2R	Two-way rmANOVA, Treatment F_1, 12_ = 0.0292, ^ns^*p* = 0.8673; Session F_10, 120_ = 3.071, **p = 0.0017; Session x Treatment: F_10, 120_ = 1.191, ^ns^*p* = 0.3038		
D1R Test	One-way ANOVA (acquisition), F_4,24_ = 9.623, ****p < 0.0001			*n* = 7
	T(1) vs T(2) (Fisher’s LSD)		*p = 0.124	
	T(1) vs T(3) (Fisher’s LSD)		****p < 0.0001	
	T(1) vs T(4) (Fisher’s LSD)		***p = 0.0001	
	T(1) vs T(5) (Fisher’s LSD)		***p = 0.0001	
	One-way ANOVA (test sessions), F_5,30_ = 1.695, ^ns^*p* = 0.1663			
	T(5) vs ET(6) (paired)		*t*_6_ = 2.152, ^#^*p* = 0.0749	
	T(5) vs ET(7) (paired)		*t*_6_ = 1.368, ^ns^*p* = 0.2202	
	T(5) vs ET(8) (paired)		*t*_6_ = 2.252, ^#^*p* = 0.0653	

f.				
Vehicle Acquisition	One-way ANOVA, F_10,60_ = 1.011, ^ns^*p* = 0.4448			*n* = 7
D1R Acquisition	One-way ANOVA, F_10,60_ = 3.283, **p = 0.0019			*n* = 7
	T(5) vs ET(6) (Fisher’s LSD)		**p = 0.0091	
	T(5) vs ET(7) (Fisher’s LSD)		**p = 0.0049	
	T(5) vs ET(8) (Fisher’s LSD)		**p = 0.0032	
	T(5) vs LT(9) (Fisher’s LSD)		**p = 0.0016	
	T(1) vs LT(10) (Fisher’s LSD)		**p = 0.0017	
	T(1) vs LT(11) (Fisher’s LSD)		**p = 0.0035	
D2R Acquisition	One-way ANOVA, F_10,60_ = 0.8263, ^ns^*p* = 0.6051			*n* = 7
Between treatments	Veh vs D1R	Two-way rmANOVA, Treatment F_1, 12_ = 4.964, *p = 0.0458; Session F_10, 120_ = 3.162, **p = 0.0013; Session x Treatment: F_10, 120_ = 2.627, **p = 0.0063		*n* = 7
		T(2) (Fisher’s LSD)	**p = 0.0014	
		T(3) (Fisher’s LSD)	**p = 0.0051	
		T(4) (Fisher’s LSD)	*p = 0.0104	
		T(5) (Fisher’s LSD)	**p = 0.0018	
	Veh vs D2R	Two-way rmANOVA, Treatment F_1, 12_ = 0.8368, ^ns^*p* = 0.3783; Session F_10, 120_ = 0.8253, ^ns^*p* = 0.6050; Session x Treatment: F_10, 120_ = 0.9407, ^ns^*p* = 0.4988		
	D1R vs D2R	Two-way rmANOVA, Treatment F_1, 12_ = 0.7011, ^ns^*p* = 0.4188; Session F_10, 120_ = 2.953, **p = 0.0024; Session x Treatment: F_10, 120_ = 2.049, *p = 0.0340		
		T(4) (Fisher’s LSD)	*p = 0.0462	
D1R Test	One-way ANOVA (acquisition), F_4,24_ = 5.751, **p = 0.0022			*n* = 7
	T(1) vs T(5) (Fisher’s LSD)		*p = 0.0211	
	One-way ANOVA (test sessions), F_5,30_ = 4.097, **p = 0.0059			
	ET(7) vs LT(10) (Fisher’s LSD)		*p = 0.0488	
	ET(8) vs LT(10) (Fisher’s LSD)		*p = 0.0326	
	ET(8) vs LT(11) (Fisher’s LSD)		*p = 0.0336	
	T(5) vs ET(6) (paired)		*t*_6_ = 2.482, *p = 0.0477	
	T(5) vs ET(7) (paired)		*t*_6_ = 1.940, ^ns^*p* = 0.1005	
	T(5) vs ET(8) (paired)		*t*_6_ = 2.020, ^#^*p* = 0.0899	

g.			
D1R Test	One-way ANOVA, F_5,30_ = 5.811, ***p = 0.0007		*n* = 7
	ET(7) vs LT(9) (Fisher’s LSD)	**p = 0.0013	
	ET(7) vs LT(10) (Fisher’s LSD)	**p = 0.0013	
	ET(7) vs LT(11) (Fisher’s LSD)	**p = 0.0013	
	ET(8) vs LT(9) (Fisher’s LSD)	**p = 0.0018	
	ET(8) vs LT(10) (Fisher’s LSD)	**p = 0.0018	
	ET(8) vs LT(11) (Fisher’s LSD)	**p = 0.0018	

h.			
Between treatments	One-way rmANOVA, F_2,18_ = 0.5478, ^ns^*p* = 0.5876		*n* = 7
	veh vs D1R (Dunnett)	^ns^*p* = 0.5074	
	veh vs D2R (Dunnett)	^ns^*p* = 0.9463	

i.			
Between treatments	One-way rmANOVA, F_2,18_ = 0.4792, ^ns^p = 0.6270		*n* = 7
	veh vs D1R (Dunnett)	^ns^*p* = 0.5441	
	veh vs D2R (Dunnett)	^ns^*p* = 0.9416	

j.			
Between treatments	veh vs D1R (unpaired, Holm-Sídák)	borders: *t*_12_ = 2.358, *p = 0.0362 middle: *t*_12_ = 5.177, ***p = 0.0007 center: *t*_12_ = 3.681, **p = 0.0062	*n* = 7
	veh vs D2R (unpaired, Holm-Sídák)	borders: *t*_12_ = 0.9039, ^ns^*p* = 0.3838 middle: *t*_12_ = 2.627, ^#^*p* = 0.0649 center: *t*_12_ = 1.821, ^ns^*p* = 0.1785	
	D1R vs D2R (unpaired, Holm-Sídák)	borders: *t*_12_ = 0.9331, ^ns^*p* = 0.3691 middle: *t*_12_ = 3.185, **p = 0.0040 center: *t*_12_ = 1.483, ^ns^*p* = 0.3011	

k.		
Between treatments	Two-way rmANOVA: Treatment F_2, 18_ = 1.139, ^ns^*p* = 0.3421 Trial F_8, 144_ = 1.900, ^ns^*p* = 0.0643, Trial × Treatment: F_16, 144_ = 0.4960, *p* = 0.9460	*n* = 7

Panels represent: **a**. Optical density, as detected by immunohistochemistry, **b**. Skilled reaching: success ratios, **c**. Skilled reaching: speed, **d**. Skilled reaching: error improvement (grasping slips), **e**. Skilled reaching: error improvement (pellet drops), **f**. Skilled reaching: time engaged, **g**. Skilled reaching: number of test pellets, **h**. Open field: distance traveled, **i**. Open field: velocity, **j**. Open field: time spent, **k**. Rotarod: latency to fall. In analyses where an ANOVA led to significant results, further comparisons were reported only when found significant (*p* < 0.05) or close to significance (*p* = 0.099, or *p* = 0.05)

*Acquisition*—vehicle, D1R or D2R antagonist injected prior to acquisition; *D1R Test*—D1R antagonist injected prior to early test of the skilled reaching task; *Med*—medial, *MedL*—medio-lateral, *MedDL*—dorso-lateral, *MedCM*—caudo-medial subfield of fastigial nucleus; *Veh- vehicle*; *T(1) – T(5)*: training sessions (acquisition); *ET(6) – ET(8)*: early test sessions; *LT(9) – LT(11)*: late test sessions in the skilled reaching task

## Results

### D1R and D2R exhibit subfield-specific expression in the rat fastigial nucleus

**Fig. 2:**
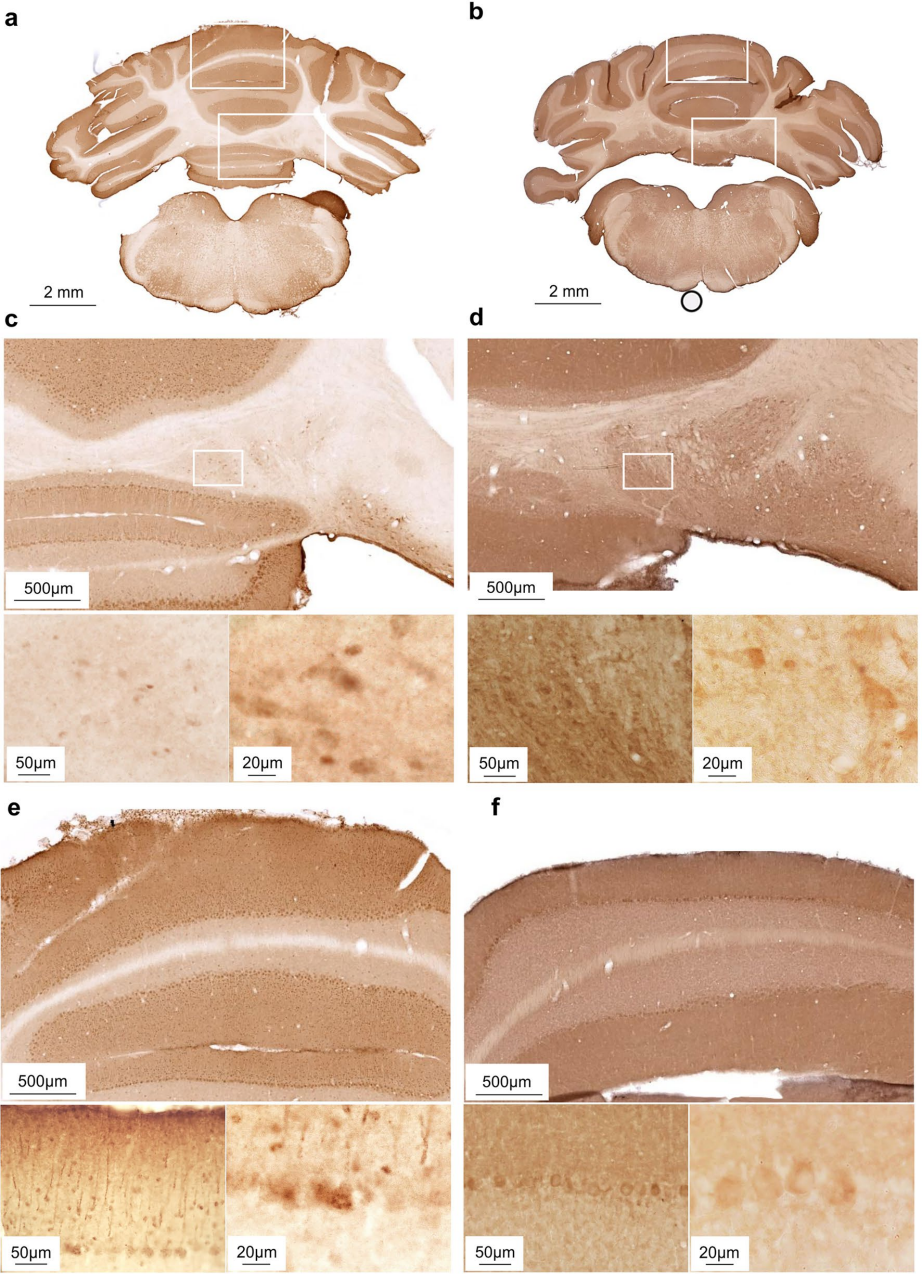
Distribution of D1R and D2R receptors within cerebellar structures. **a** Overview of D1R immunostained coronal rat brain slice including caudomedial FN subfield. **b** Overview of D2R immunostained coronal rat brain slice including medial FN subfields. **c, e** High magnification selections from either FN (**c**) or lobule VI of the cerebellar cortex (**e**) from the D1R immunostained brain slice in **a**. **d, f** High magnification selections from either FN (**d**) or lobule VI of the cerebellar cortex (**f**) from the D2R immunostained brain slice in **b**. White rectangles mark selections for higher magnification images. Scale bars for each image are mentioned in the lower left corner for each panel

First, we examined the expression of D1R and D2R in the rat FN using an IHC approach. For this, we scrutinized receptor expression in the medial, lateral, dorso-lateral, and caudo-medial fastigial areas. We observed that the expression of both D1R and D2R varies significantly across FN subfields when compared within each receptor group (Fig. 1c, d; Table 1a; D1R, ****p < 0.0001, D2R, ***p = 0.0003, *n* = 5). Optical density measurements of receptor expression levels, for either DA receptor measured, indicated that the medio-lateral area of the FN exhibits the highest DA receptor density, while the caudo-medial expresses the least (Fig. 1c, d). A qualitative inspection of the receptor labeling (as shown in Fig. 2) indicated that both DA receptors are localized in FN cell bodies. As for the other cerebellar areas, our results (Fig. 2) confirmed previous findings by others regarding the presence and distribution of D1R and D2R in the cerebellar cortex layers. They are mainly present in molecular and Purkinje layers for both receptors, and additionally in the granular layer for D2R (Martres et al. 1985; Camps et al. 1990; Ricci et al. 1995, 1996; Barili et al. 2000; Flace et al. 2021; Locke et al. 2018), and D1R is present in the dentate nucleus (Locke et al. 2018) and along the processes of the Bergmann glia innervating the cerebellar molecular layers (Cutando et al. 2021; Li et al. 2023) as seen in Fig. 2e. Interestingly, in FN we see a similar expression pattern of D1R and D2R as in the dentate nucleus, i.e. while D1R is more pronouncedly expressed at the level of the neuronal cell bodies, D2R is also expressed in high levels in neuronal processes (Flace et al. 2021).

### Pharmacological antagonism of the FN D1R, but not D2R, modulates reaching skill acquisition

**Fig. 3:**
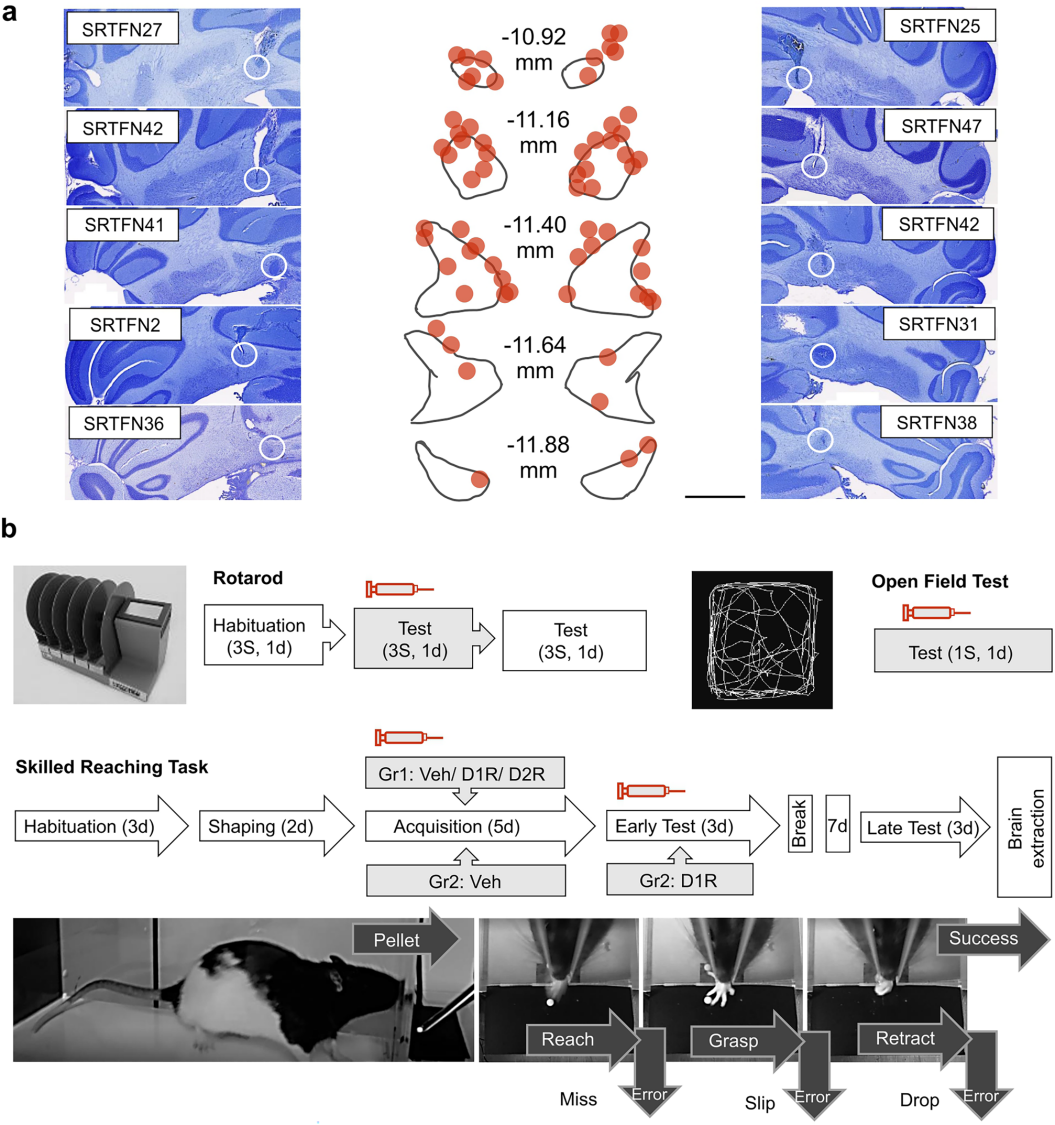
Framework of behavioral experiments. **a** Localization of vehicle or DA receptor antagonist injection sites. Left and right panels represent examples of cresyl violet stained sections where the cannula tip is marked by white circles. The middle panel is a reconstruction of cannulas placement withing the FN (according to Paxinos and Watson 2005) for each animal included in the study. The red dots represent the cannula tip locations. Coordinates, expressed in mm, represent anterior to posterior positioning to bregma. **b** Experimental procedures for each of the three behavioral tasks used. In the upper left panel, the rotarod protocol is illustrated, while in the right upper panel, the protocol for the open field test is shown. The middle panel illustrates the skilled reaching task protocol (events/day). The lower panels show the training events (forearm reach, paw grasp, arm retraction) including errors aspects (reach miss, grasp slip, pellet drop during retraction) that need to be overcome before the animal successfully completes a skilled reaching maneuver. Syringe symbol and gray background indicate the sessions that included a prior treatment with vehicle or DA ligand. S = number of sessions, d = number of days, Gr1 = group 1, Gr2 = group 2, Veh = vehicle treatment, D1R/D2R = dopamine antagonist treatment. See also, elevated plus-maze schema in supplementary data (Fig. S1a)

Next, we trained the rats in a skilled reaching task during pharmacological antagonism of either D1R or D2R, enabled by means of bilateral microinjection into the FN (Fig. 3). Despite cannulas tip placement variability within the FN (Fig. 3a), we did not detect outliers in the behavioral results of our experimental groups.

**Fig. 4:**
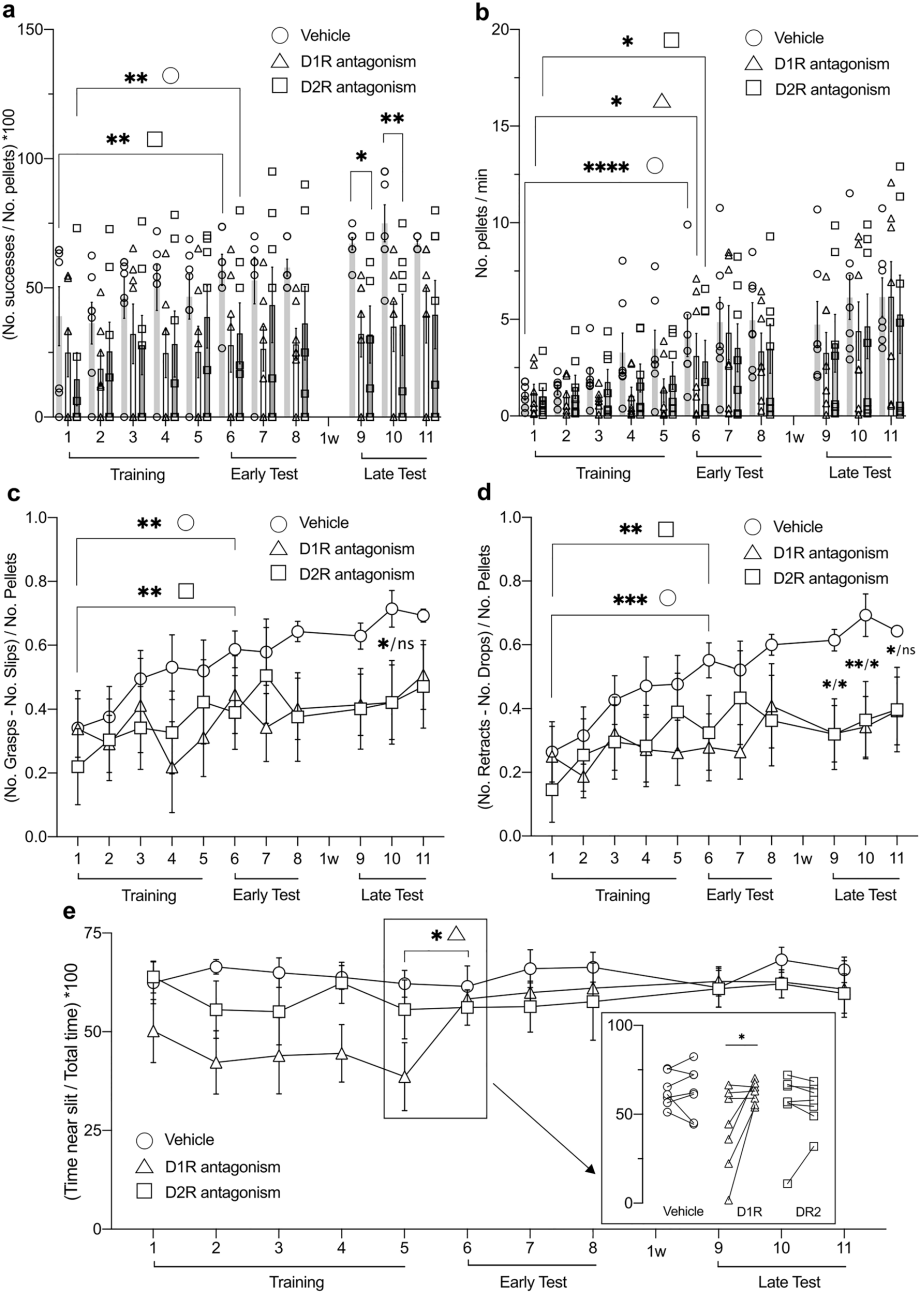
Skilled reaching learning is modulated by pharmacological antagonism of D1R and D2R in the FN. **a** Success ratio across all trials and treatment groups. Learning progression (Training 1–5) is impaired in D1R antagonist-treated animals, but progresses steadily for vehicle and D2R antagonist-treated groups (significant improvement from sessions 1–6). Performance was lower than in controls, one week after the initial testing for both antagonist groups. **b** The speed of pellet reaching is significantly higher when the first training day is compared with the first early test day in all treatment groups (session 1 vs 6). **c-d** Error improvement is significantly higher when the first training day (1) is compared with the first early test day (6) in all treatment groups, in the case of number of slips (**c**) and drops (**d**). Significantly lower rates were, however, found during late performance testing, when antagonist-treated and control groups were compared. **e** Overview of the percentage of time spent near the pellet access slit, relative to the total time spent in the chamber. D1R antagonist-treated animals gradually spent less time in front of the slit, an effect that was reversed when antagonist treatment was stopped (starting with the first day of early testing, session number 6). The inset (rectangle on the right side of the graph) provides a comparison of individual data points between last day of training (session 5) and first day of early test (session 6), to illustrate how the D1R antagonist treatment affected task engagement level. Group symbols: open circle = vehicle; open triangle = D1R antagonist; open square = D2R antagonist. For all comparisons, *p < 0.05, **p < 0.01, ***p < 0.001, ****p < 0.0001 (all t-test); *n* = 7. Training (1–5) = training sessions (acquisition phase); Early Test (6–8) = early test sessions; 1w = one week interval between early and late testing sessions; Late Test (9–11) = late test sessions. For further statistics between treatments, see Results sections and Table 1b-g

The vehicle-injected controls showed a significant increase in their success ratios across the acquisition sessions (Fig. 4a; Table 1b; ***p = 0.0001, *n* = 7). However, when the D1R antagonist (SCH 5.94 µg/hemisphere) was administered 30 min prior to each acquisition session, no learning progression was evident during acquisition sessions (Fig. 4a; Table 1b, ^ns^*p* = 0.2164, *n* = 7). By contrast, the D2R antagonist group (remoxipride, 10 µg/ hemisphere) demonstrated a progressive acquisition of the reaching skill (Fig. 4a; Table 1b, **p = 0.0024, *n* = 7), with no significant differences evident compared to controls (Fig. 4a; Table 1b, treatment factor: ^ns^*p* = 0.1043). Nonetheless, some significant differences were identified in the late test sessions, whereupon D2R antagonist-treated animals showed a significantly weaker performance compared to controls for the same sessions (Table 1b, vehicle vs D2R: LT(9), *p = 0.0156; LT(10), **p = 0.0071). Error improvement rates significantly increased across the acquisition sessions (Fig. 4c, d), for both (grasping) slips (**c**) and (pellet) drops (**d**), in the vehicle and D2R antagonist groups, but not the D1R antagonist group (Table 1d-e, Slips: vehicle ***p = 0.0003, D1R ^ns^*p* = 0.2578, D2R **p = 0.0010; Drops: vehicle ****p < 0.0001, D1R ^ns^*p* = 0. 2238, D2R **p = 0.0013; *n* = 7). Similar to the success ratio, in the late phase testing, both antagonist groups showed poorer performance than controls in the error rates (Fig. 4c, d; Table 1d-e**;** LT (9–11)). In other words, D1R antagonism hindered task acquisition, whereas D2R antagonism impaired the late optimization of task efficacy.

As the D1R antagonist had a strong effect on the acquisition of reaching skill, we subsequently tested the effect of D1R antagonism on reaching skills that had already been successfully acquired.

### D1R antagonism in FN does not impair established reaching skills, but facilitates reach-to-success errors

**Fig. 5:**
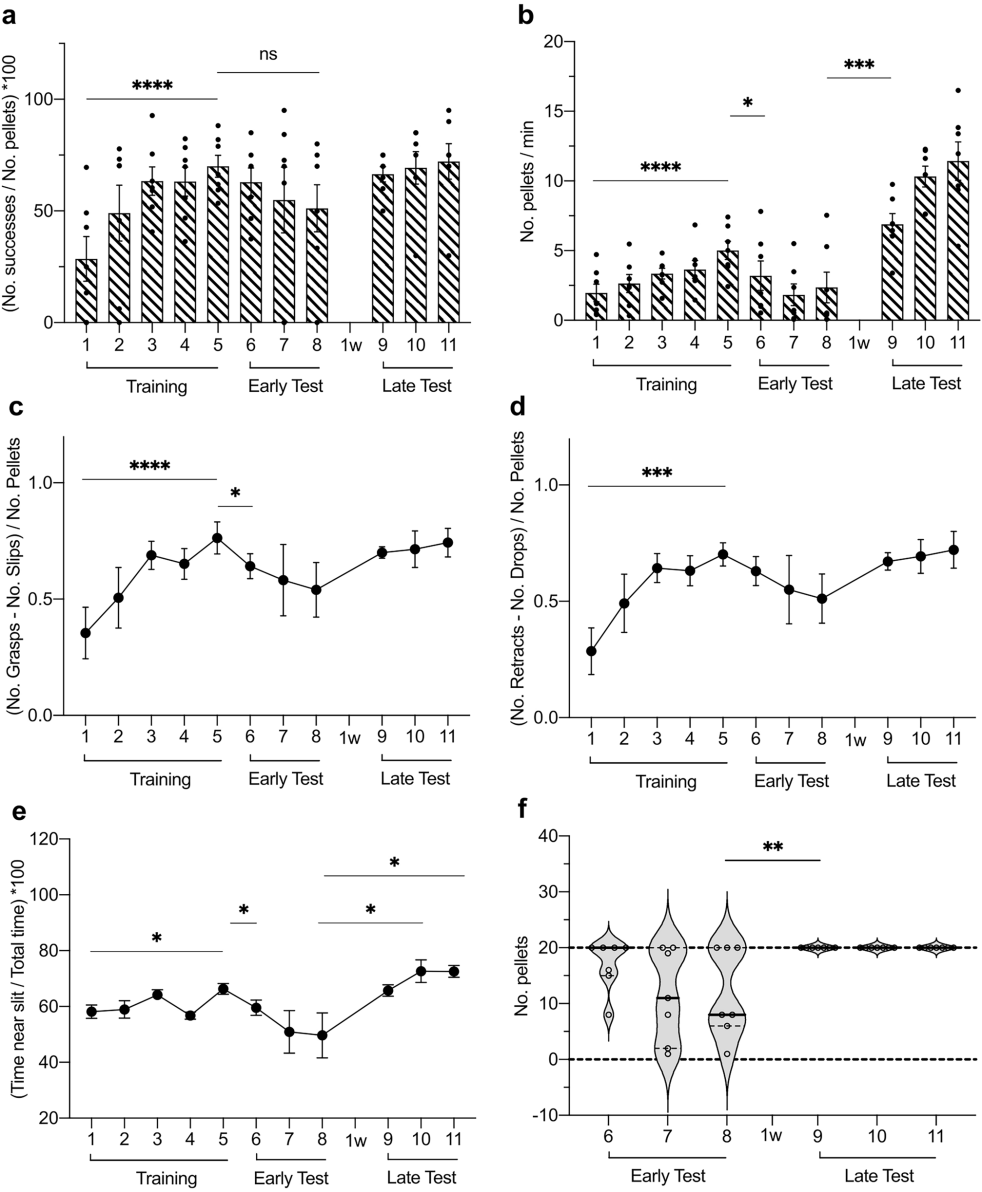
Antagonism of FN D1R after reaching skill acquisition does not affect the success ratio but modulates error rates and task engagement. **a** Success ratio increases along training events (first versus fifth training session) and is not significantly diminished by D1R antagonist infusion after the reaching skill was acquired (fifth training session versus each early test sessions, and third early test sessions versus all late testing sessions). **b** Speed of pellet reaching increases during the acquisition sessions (first versus last training session), but it is significantly altered by D1R antagonist treatment (last training session versus each early test sessions, and third early test sessions versus all late testing sessions). **c-d** Error improvement rates for either slips (**c**) or drops (**d**) increase during the acquisition sessions (first versus last training session) but show a decrease after ligand infusion (significant only for slip errors, last training session versus first early test session). **e** The percentage of time spent near the pellet access slit increases during acquisition (first versus last training session), decreases with D1R antagonist treatment (last training session each early test sessions) and then returns to vehicle levels 7 days after antagonist treatment (last early test session versus each later test sessions). **f** This phenomenon is also reflected by the number of pellets approached during the test sessions, whereby much lower numbers are evident during the D1R antagonist sessions compared to vehicle treatment sessions (e.g., last early test session versus first late test session). For all comparisons, *p < 0.05, **p < 0.01, ***p < 0.001, ****p < 0.0001, *ns* = not significant, *n* = 7 (*t*-test). Error bars represent ± SEM. Training (1–5) = training sessions (acquisition phase); Early Test (6–8) = early test sessions; 1w = one week interval between early and late testing sessions; Late Test (9–11) = late test sessions. For further statistics, see Results sections and Table 1b-g

For this experimental group, the D1R antagonist was applied before each of the three testing sessions. We observed that treatment with the antagonist *after* skilled reaching had been acquired had no effect on the successful reaching ratio, as performance was at similar levels to that seen on the last day of training. Here, at least 50% of the reaches were successful (Fig. 5a; Table 1b, T(5) vs ET(6–8), ^ns^p > 0.05). The error improvement rate significantly increased during acquisition, as expected (Fig. 5c, d, Table 1d-e, Slips: ***p = 0.0003, Drops: ***p < 0.0001, *n* = 7). However, a significant reduction in error improvement rate was seen when comparing the (grasping) slips rate of the last day of acquisition with the first day of the test session following D1R antagonist administration (Table 1, Slips: T(5) vs ET(6), *p = 0.0483, *n* = 7).

### Antagonism of D1R in FN lowers engagement in skilled reaching and alters open field behavior without affecting motor function

Given the role of DA in modulating motivation for action (Schultz 2007), we assessed whether state-dependent factors contributed to the effects of D1R antagonism on learning behavior. For this, we first examined the engagement of the animals in the task. This was based on the consideration that the skilled reaching task we implemented in this study is goal-oriented. Even though D2R antagonism had no appreciable effects on learning, we assessed the contribution of this receptor, as well as D1R, to state-dependent effects, as it is not unreasonable to expect that the receptor might modulate motivation independently of learning.

In the first experiment, vehicle, D1R, or D2R antagonists were applied prior to the commencement of each acquisition session. Here, we tested the speed at which the animal reached the pellets with a forepaw during the acquisition and in the subsequent test sessions. The speed of reaching the pellets significantly increased from day 1 of training, T(1), through the first day of the test, ET(6), in vehicle-treated controls and the groups treated with either D1R or D2R antagonists (Fig. 4b; Table 1c, vehicle: ****p < 0.0001, D1R *p = 0.0323, **p = 0.0034, *n* = 7). No significant differences in the speed of reaching the pellets were found when the treatment groups were compared with one another (Table 1c).

We then measured the time an animal spent in front of the pellet access slit (i.e. time spent within a distance of 5 cm from the slit), with its nose oriented towards the wall that contained the slit. Here we observed that the D1R antagonist group spent less time in front of the slit compared to the vehicle group (Fig. 4e; Table 1f, treatment factor: *p = 0.0458, *n* = 7), with significant differences in acquisition sessions T(2) to T(5) (Table 1f, vehicle vs D1R: *p* < 0.05, *n* = 7). By contrast, no significant differences occurred in the time spent close to the slit when vehicle and the D2R antagonist-treated animals were compared (Fig. 4e; Table 1f).

In the second experiment, the D1R antagonist was infused prior to the early test sessions. We first confirmed that the speed of approaching the pellets increased along the acquisition sessions, as was found in the previous experiment (Fig. 5b**; **Table 1b**,** T(1) vs T(5): ****p < 0.0001, *n* = 7). However, following D1R antagonist treatment prior to the early test (ET) sessions, the speed significantly decreased (Fig. 5b**; **Table 1c, T(5) vs ET6-8: *p* < 0.05, *n* = 7). Thus, D1R antagonism after task acquisition impaired reaching performance in the memory retrieval test, although it left reaching success ratio unaffected. Interestingly, the pellet approach speed in the late testing (LT) sessions, one week after D1R antagonist treatment, not only recovered, but rather significantly exceeded the speed level of the last acquisition (T) session (Fig. 5b**, **Table 1c, T(5) vs LT(9–10); *p* < 0.0001, *n* = 7). This indicates that memory of the skill was not impaired in the early test sessions, but rather the task engagement was transiently altered by the presence of the antagonist during these sessions. Notably, during the late test phase, the rats that received the D1R antagonist prior to early testing, exhibited speed values that were almost double the values measured in the group that was injected prior to the acquisition phase (Fig. 4b vs 5b; average speed with standard deviation in the third session of late test in Fig. 4b: mean = 6.153 pellets/min (SD 2.644), in Fig. 5b: mean = 11.427 pellets/min (SD 3.656)). This suggests that D1R-antagonist application, during the early consolidation phase of the skill, reinforces task learning, perhaps by placing more demands on effortful participation. Subsequently, once antagonist levels had declined, the motivation to engage in the task increases, as seen in the high-speed values of the late testing sessions (Fig. 5b).

The D1R-treated animals in this experiment spent significantly less time in front of the pellet access slit (Fig. 5e; Table 1f, T(5) vs ET(6): *p = 0.0477,* n* = 7) and approached fewer pellets at test (Fig. 5f; Table 1g, ET(8) vs LT(9), **p = 0.0018,* n* = 7) when the antagonist was delivered prior to each early test session. This is in line with the findings from the first experiment, where we observed that treatment with the D1R antagonist prior to the acquisition sessions lowered task engagement (Fig. 4b) and time spent near the slit (Fig. 4e).

**Fig. 6:**
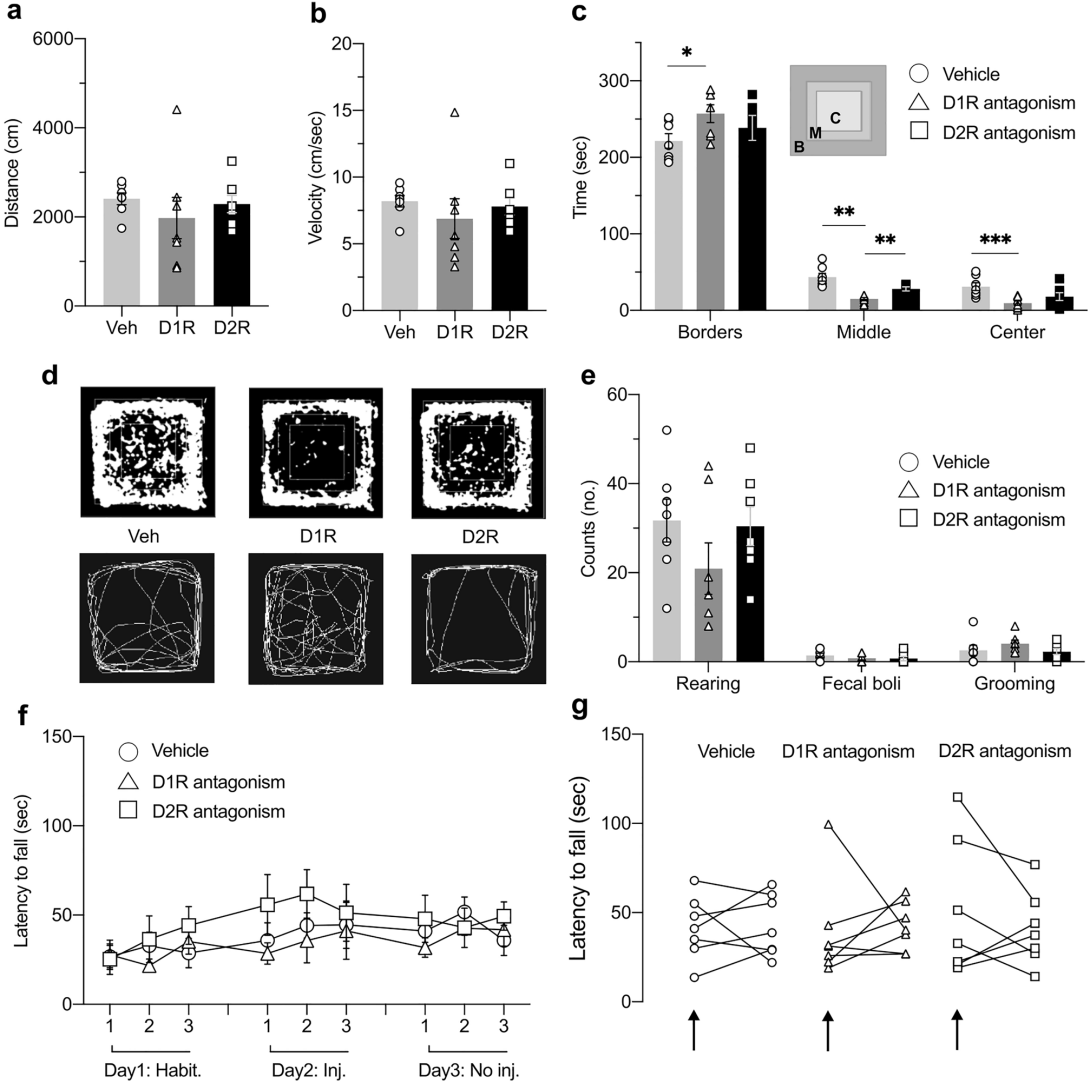
Motor coordination and locomotion are not affected by dopamine antagonist treatment of the fastigial nucleus, but thigmotaxis is increased by D1R antagonism. **a-b** Ambulation, measured either as total distance traveled (**a**) or velocity (**b**), calculated for the 5 min of open field exploration, was not affected by infusing dopamine antagonists into the fastigial nucleus (FN) prior to the test. **c** The distribution of time spent in open field locations indicates that the D1R antagonist-treated animals preferred to explore the borders more than the center of the arena. Behavior in the D2R antagonist group was not statistically significant compared to controls but was significantly different from D1R antagonist-treated animals (inset: C—center, M—middle, B—borders). **d** Exploration in the open field arena following vehicle or dopamine antagonist treatment. The maps in the upper panel illustrate the mean exploration coverage of the arena by each treatment group (*n *= 7). In the lower panel, track samples from each treatment group illustrate locations covered during open field exploration. **e** Counts of rearing bouts, fecal boli, and grooming events recorded in each treatment group. No significant differences were found between groups for either of the counted events. **f-g** Rotarod results. Panel **f** shows data from all three sessions; habituation during session 1, test with prior treatment during session 2, test with no treatment during session 3 (each test includes three trials, 1–3). Panel **g** shows the individual data points used to compare the means of test trials conducted in the presence of treatment versus no treatment. For each treatment group, the first dataset represents the session with prior injection (indicated by black arrows), while the second dataset represents the session without prior injection. No significant differences were found within, or between groups for either session. Habit. = habituation sessions (Day 1, at 5 rpm); (No) Inj. = injected (day 2) or not injected (Day 3) sessions (increasing speed, 4 to 40 rpm). Group symbols: open circle = vehicle; open triangle = D1R antagonist; open square = D2R antagonist. For all comparisons, *p < 0.05, **p < 0.01, ***p < 0.001, *n* = 7 (*t*-test). Veh: vehicle; D1R: D1R antagonist; D2R*:* D2R antagonist

To assess whether changes in motor function influenced task engagement, we examined open field and rotarod test behavior to scrutinize general ambulation and motor coordination, balance, and grip strength. Distance and velocity measurements for all treatment groups indicated that no significant alterations were caused by DA receptor antagonism (Fig. 6a, b; Table 1h-i, Distance: ^ns^*p* = 0.5876, Velocity: ^ns^*p* = 0.6270; *n* = 7). Furthermore, no significant differences in rotarod latencies were found in any session when the three treatment groups were compared, or when performance *within* each treatment group was assessed (Fig. 6f, g; Table 1k, treatment factor: ^ns^*p* = 0.3421, *n* = 7).

Altogether, these results suggest that D1R antagonism in the FN may modulate the task engagement behavior of the rats, without increasing anxiety levels, and thus, may affect their *motivation* in approaching the pellets.

To test this possibility further, we also looked at the distribution of time spent in different locations of the arena in an open field test, to discriminate the animal’s thigmotactic behavior, that reflects a rat’s innate proclivity to stay near environment borders (Treit and Fundytus 1988). Thigmotaxis has been proposed to serve as an indicator for anxiety (Prut and Belzung 2003), changes in cognitive strategies (Hostetter and Thomas 1967), or idiothetic information processing (Goddard et al. 2008). A comparison of the time spent in the borders, middle area, and center of the arena, across all three treatment groups (Fig. 6c), indicated that D1R antagonist injection into the FN alters open field behavior in the rats. The D1R antagonist group spent significantly less time in the center, or the middle zone of the arena compared to vehicle treated animals, and consequently spent more time at the arena borders (Table 1j, vehicle vs D1R, Center: **p = 0.0062, Middle: ***p = 0.0007, Borders *p = 0.0362,* n* = 7). In addition, this group spent less time in the middle zone than the D2R antagonist group (Table 1j, D1R vs D2R, Middle: **p = 0.0040,* n* = 7). Moreover, the D1R antagonist-treated group crossed the center area less often than the vehicle group (Fig. S1b, Table S1). No significant differences were found when comparing the time spent in each area between the vehicle and the D2R groups (Fig. 6c). No differences in biomarkers for anxiety were detected in the three treatment groups, such as number of fecal boli excreted, and counts of rearing and grooming bouts (Fig. 6e). The time spent either in the open or closed arms of an elevated plus maze did not differ between groups (Fig. S1a, Table S1). These data suggest that D1R antagonism altered the animals’ exploration strategy in the open field and the motivation to explore, without raising anxiety levels.

## Discussion

In this study, we describe a novel role for the FN in the acquisition and modulation of goal-directed behavior, based on skilled reaching. Effects are mediated by DA receptors and specifically target task acquisition and task engagement, but not motor behavior. Scrutiny of DA receptor expression revealed that D1R and D2R are expressed in the FN of adult rats. Expression is distributed in the respective FN subfields, whereby the highest receptor density in the FN is found in the medial lateral subfield, and the lowest is found in the caudo-medial part. The FN contribution to skilled reaching was primarly mediated by D1R.

### The fastigial nucleus contributes to memory of goal-directed skill reaching and effects are dopamine-receptor dependent and motivation-related

In this study, We examined the acquisition of skilled reaching in a goal-directed task that allowed the animals to learn how to effectively grasp a food reward. Under vehicle conditions, across five days of acquisition, rats successfully learned to reach single-pellets by skillfully approaching them by inserting their forepaw through a narrow wall slit. The success ratio increased significantly from the first to fifth day of acquisition, along with an improvement in (grasping) slip, and (pellet) drop, error rates. The time spent engaged with the task remained constant along all task sessions. D1R antagonism prevented task acquisition by modulating the motivation to engage in a goal-directed task and without affecting motor performance, but by modulating the motivation to engage in a goal-directed task. The D2R antagonism during the acquisition only affected late performance.

Participation in goal-directed behavior has been ascribed to the cerebellum (Babayan et al. 2017; Verschure et al. 2014) and the deep cerebellar nuclei (DCN) (Callu et al. 2013; Xiao et al. 2018). Projections from the murine DCN to the striatum have been described that are glutamatergic and are proposed to mediate these effects (Xiao et al. 2018). Whereas operantly conditioned forelimb movement is supported by the interpositus (Milak et al. 1997), goal-directed behavior may be modulated by both interpositus and the FN (Xiao et al. 2018). However, another study reported that neither the dentate nor interpositus nuclei are needed for the learning of complex forelimb movement (Wang et al. 1998), pointing to a specific role for the FN in the learning of skillful limb movement. Although the study by Xiao and colleagues indicated a role for the FN in goal-directed behavior, inactivation of the DCN was only conducted unilaterally, and discrete targeting to discriminate the roles of the FN versus interpositus nucleus was not conducted. In our study, we conducted bilateral localized targeting of DA receptors in the FN and show that this structure supports both the acquisition and consolidation of goal-directed skilled reaching.

The abovementioned effects on goal-directed behavior were mediated most particularly by D1R. Here, D1R antagonism prior to acquisition sessions, prevented skill acquisition, as seen both in the stagnant success ratio and significant prevention of error reduction. The effects of D1R antagonism on task acquisition are consistent with reports of a role for this receptor in memory acquisition and consolidation. For example, it has been reported that a D1R antagonist, infused into the motor cortex, impairs goal-directed motor skill learning and long-term cortical plasticity (Molina-Luna et al. 2009). D1R are also required for hippocampal-dependent learning and memory, and related synaptic plasticity (Hansen and Manahan-Vaughan 2014), as well as for prefrontal cortex-dependent working memory (Takahashi et al. 2008). We observed that the time spent by D1R antagonist-treated animals in front of the pellet access slit was significantly lower than that of the vehicle group in the experiments where antagonism was implemented either prior to, or after, acquisition. Thigmotaxis increased in D1R antagonist-treated animals, in the absence of anxiogenic effects. These results suggest that D1R antagonism within the FN reduces task engagement. Findings are in line with studies conducted in D1R knockout (KO) mice that show lower performance in motivation-driven motor skill acquisition (Nakamura et al. 2014). An interesting property of the D1R antagonist effects in the FN was that we found that subsequent retrieval of the learned skill, one week after D1R antagonist-mediated impairment of early retrieval, was not impaired. Rather skilled reaching was significantly improved, as seen in the speed of reaching. This suggests that although task engagement was reduced during early testing (and thus early consolidation) when the D1R antagonist was present, subsequent consolidation of the skilled learning experience must have occurred that served to improve subsequent performance one week later. This may have been mediated by DA acting on D2R.

D2R antagonism within the FN was much less effective at modulating skilled learning than D1R antagonism. Here, although no effects were detected during task acquisition or early retrieval testing, lower task performance was observed in the late test sessions, compared to controls. This suggests that D2R antagonism affects late consolidation, or indeed post-consolidation of task acquisition. Similar to our finding, Nakamura and colleagues reported lower performance in later phases post-acquisition of a motor skill acquisition task in D2R KO mice (Nakamura et al. 2014). Taken together our findings suggest that D1R is more involved in the initial learning of the skill, whereas D2R is involved in task consolidation.

### Dopaminergic projection into FN

We specifically targeted DA receptors in the FN by local infusion of D1R and D2R antagonists. The question arises as to how DA reaches these receptors under natural circumstances. Although the VTA was shown to send projections to the deep cerebellar nuclei (Ikai et al. 1994), no VTA dopaminergic terminals have been found to project to the FN specifically (Ikai et al. 1992, 1994). A more recent hypothesis points to the locus coeruleus as the potential dopaminergic source to the vermis, as recent findings demonstrated noradrenaline (NA) and dopamine co-release in other brain areas (Kempadoo et al. 2016; Beas et al. 2018) and the locus coeruleus has also been shown to modulate D1R in the vermal granular layer (Canton-Josh et al. 2022). Thus, projections from locus coeruleus into the FN may comprise the endogenous source of DA for the effects we detected in our study. Considering the important role of vermis in affective function modulation (Jackman et al. 2020) and its reciprocal projections to the FN (Fujita et al. 2020), it is possible that the motivational effects we reported here might be controlled via these projections.

### Potential FN projections modulating goal-directed behavior

The next consideration is how DA receptors of the FN can modulate skilled reaching and the learning of goal-directed behavior. As mentioned earlier, targeting of DCN projections to the striatum modulates goal-directed behavior (Xiao et al. 2018) and it is thinkable that DA acting on its receptors in the FN can alter output to this structure. Midbrain dopamine neuronal projections have been shown to modulate reach kinematics, related coordination movements, and also affective-state related aspects (Bova et al. 2020; Leemburg et al. 2018). One possibility is that the FN may modulate the nigrostriatal bundle to affect goal-directed behavior, as it was recently reported that FN projections to VTA are involved in reward processing and social behavior (Carta et al. 2019), while projections from FN to the substantia nigra are involved in movement initiation, vigor, and reward processing (Washburn et al. 2022). In this bigger circuitry, the role DA receptors in the FN might play is that of predicting reward error, considering the decrease in the task engagement we observed following D1R antagonism. This interpretation is supported by the anatomical connections between the DCN and the basal ganglia described above. It is also possible that the dopaminergic signal, that reaches the FN from the locus coeruleus, affects reward prediction by modulating valency weighting, a role which has been proposed for the locus coeruleus in other subcortical structures (Poe et al. 2020).

Another potential mechanism of action underlying the FN modulation of goal-directed behavior could be that DA receptors in the FN modulate its long-range glutamatergic projections, such as the projections to the ventrolateral periaqueductal gray (vlPAG), or to the striatum (Xiao et al. 2018). The vlPAG plays an important role in freezing behavior in rodents (Frontera et al. 2020; Vaaga et al. 2020) and triggers akinetic mutism and apraxia in humans (McAfee et al. 2022). Glutamatergic FN projections to the vlPAG have been demonstrated in rodents (Vaaga et al. 2020; Frontera et al. 2020). Alternatively, FN DA receptors could modulate the action of the GABAergic neurons projecting to the inferior olive, knowing that D1R are expressed in these neurons in other cerebellar nuclei (Locke et al. 2018). Finally, another possibility is that DA receptors in the FN can affect the output action of the FN glycinergic neurons projecting to the vestibular and reticular nuclei given that these projections, originating in the rostral FN, mediate adjustment of posture and balance, autonomic function, which are crucial for goal-directed action (Bagnall et al. 2009).

### A subfield distribution of dopamine receptors in the fastigial nucleus

In this study, we identified a difference in the distribution of DA receptors across the FN. We observed that the medial lateral part of the FN expresses the highest amount of D1R and D2R, while the caudo-medial part, exhibits the lowest expression of the four FN subfields. Based on the reconstruction of the injection sites of the antagonists in the behavioral studies, we estimated that more of the medial and rostral parts were targeted by antagonist treatment than the caudal part of the structure. This raises an interesting aspect considering that studies in primates and other mammals reported that the rostral FN mainly processes vestibular information (Brooks and Cullen 2013), while the caudal part is more engaged in processing oculomotor information (Quinet and Goffart 2005). In other words, targeting DA receptors in the rostral FN may modulate learning behavior by influencing idiothetic information processing by the FN. In line with this, mono- or di-synaptic connections of the FN with non-motor areas such as the prefrontal cortex or the VTA have been described (Fujita et al. 2020), pointing to a potential role of this structure in modulating cognitive processes and affective states. The qualitative inspection of our DA receptors staining indicates a different localization of the two receptors in the FN, namely that DIR are predominantly expressed in the nuclear cell bodies, while the D2R are expressed both at the somatic level and in cell processes, confirming similar findings by others in the rat and human dentate nucleus (Locke et al. 2018; Flace et al. 2021). We also observed D1R expression along the processes of Bergmann glia in the FN. Similar expression has been reported by others for the cerebellar cortex (Li et al. 2023). Interestingly, conditional knockout of D1R in cerebellar Bergmann glia results in impairment of locomotor activity and social interactions (Li et al. 2023). We did not detect effects on locomotion in the presence of local D1R antagonism in the FN, which would suggest that Bergmann glia that are locally present in the FN may not regulate this behavior. Others have also reported a lack of effect of D1R antagonism (via SCH23390) on Bergmann glia function in the vermis and lateral lobes of the cerebellum, however (Cutando et al. 2021) and have shown than in these structures the cells are regulated by noradrenaline acting on beta-adrenergic receptors. Thus, the question remains, as yet, unresolved as to whether Bergmann glia contribute to goal-directed behavior that is regulated by the FN.

### Motor function is not affected by dopaminergic receptors in the FN

When studying cerebellar function and dopaminergic signaling, it is important to discriminate between motor and nonmotor effects. DA plays an important role in modulating motor function as shown, for example, in motor impairments triggered by the degeneration of DA neurons of the substantia nigra in Parkinson’s disease (Mamelak 2018). The modulation by DA of motor skill learning has also been documented under normal physiological conditions in various species (Wood 2021). We used rotarod and open field tests to assess if general motor coordination and ambulatory capacity were affected by DA receptor antagonist infusion. Our results confirmed that the DA receptor antagonists, in the doses used, did not affect motor coordination, velocity and distance traveled. Furthermore, our finding that pellet reaching speed was only modulated when the D1R antagonist was applied *after* the skill was acquired, indicates that this was due to changes in task engagement, rather than motor dysfunction.

Studies that examined DA receptors in the FN, and their function, are lacking. However, a few studies looked at this in the cerebellar cortex areas of the vermis, which represents the main output structure to the FN. Here, it was shown that selective D3 receptor agonism significantly decreases locomotor activity in rats (Barik and Beaurepaire 2005), whereas microinjection of D1R and D2R antagonists into vermis cerebellar lobules 5–6 of mice do not alter motor control and motor learning (Guilherme and Gianlorenço 2021). These reports do not contradict, but rather align with our results that showed that D1R and D2R antagonism in the FN did not alter motor function.

Our results in the skilled reaching task, where a decrease in engagement levels was seen after D1R antagonist treatment, together with our findings in the open field test, where the D1R antagonist group spent less time in the center, point to a regulation of behavioral affect by D1R in the FN. In the open field, D1R antagonist-treated animals preferred to remain nearer the arena walls. Increased thigmotaxis has been proposed as a behavioral biomarker for increased anxiety in rodents (Treit and Fundytus 1988; Prut and Belzung 2003). Studies have reported D1R involvement in fear signaling as indicated by prolonged fear responses after fear conditioning in mice lacking these receptors (El-Ghundi et al. 2001), or following infusion with a D1R antagonist into the amygdala (Guarraci et al. 1999). However, during the open field exploration following local antagonism of DA receptors in the FN, we did not observe evidence of fear in our rats as shown by the assessment of anxiety-related behaviors, namely, the number of fecal boli (Ren et al. 2012), rearing, and grooming (Fan et al. 2011; Masood et al. 2003). This suggests that the increased thigmotaxis caused by D1R antagonism was not related to anxiogenic effects. This was also supported by results from the elevated plus maze experiment (see supplementary data), where no difference in the time spent in open or closed arms was detected between the treatment groups.

An alternative viewpoint on thigmotaxis is that it reflects the natural proclivity of rodents to stay near the borders of an environment (Barnett 1963) and thus, may reflect an innate exploration strategy. In line with this, others have shown that lesions of the hippocampus enhance thigmotaxis and impair spatial learning (Hostetter and Thomas 1967), suggesting that in the absence of effective information encoding, thigmotaxis persists because the environment has not yet been learned. The processing of idiothetic information is a fundamental element of spatial navigation (Draht et al. 2017) and disrupting vestibular information needed for directional mapping also disrupts head direction cell behavior (Brown et al. 2002; Clark and Taube 2012). Furthermore, lesions of the vestibular system increase rodent locomotion. Bearing in mind that the FN processes vestibular information and relays it to subcortical and cortical regions (Fujita et al. 2020) one possible interpretation of our results is that D1R antagonism in the FN alters idiothetic cue processing during spatial navigation in the open field in rats. Given the postulated role of the FN in modulating affect and motivated behaviors in rats (Berntson and Torello 1980; Helgers et al. 2020; Berntson and Schumacher 1980; Al-Afif et al. 2019), and the known role of D1R in this process in general (de la Mora et al. 2010), it is also possible that D1R antagonism reduced task engagement and motivation. Our interpretation that D1R antagonism affected motivation, but not anxiety, was also supported by the reduced tendency of antagonist-treated animals to cross the center area in the open field test. This behavior was akin to that reported in animals that underwent lesions of dentate nuclei of the DCN, where a hedonic and purposive motivational reduction was also confirmed (Bauer et al. 2011; Peterson et al. 2012). Furthermore, interactions between the cerebellum and the basal ganglia have been described whereby different cerebellar projections may influence either reward-based-learning, or movement vigor (Yoshida et al. 2022). However, the specific role of dentate nucleus D1R and D2R for this function has not yet been clearly demonstrated (Locke et al. 2018).

Finally, it is important to consider the limitations of our study. The D1R antagonist used here (SCH23390), shows affinity for the 5-HT2A receptor, although this is approximately ten-fold lower than the affinity for the D1R (Ekelund et al. 2007) and also acts as an agonist for 5-HT2C and 5-HT1C receptors (Briggs et al. 1991; Skarsfeldt and Larsen 1988; Taylor et al. 1991; Benaliouad et al. 2011; Millan et al. 2001). In the rat fastigial nucleus, the expression of only 5-HT2A receptors has been reported (Zhang et al. 2014). By contrast, 5-HT1 receptors were reported to be only localized in the lateral cerebellar nuclei of the DCN (Pazos and Palacios 1985). Therefore, influence of the FN by SCH23390 action on serotonergic modulation can be assumed to be limited to a low-affinity modulation of the 5-HT2A receptor. However, activation of 5-HT2A receptors results in a facilitation of motor control performance in the rotarod and balance beam, and receptor antagonisms results in attenuation of this performance (Zhang et al. 2014). Therefore, we conclude that the main effects we saw when using SCH23390 were mainly driven by action on DA receptors.

The D2R antagonist we used (remoxipride) has been reported to have an affinity for sigma receptors (Wadworth and Heel 1990). These have been reported to be expressed in the DCN, including FN (Hohmann et al. 1992). Few studies address their role in learning-related processes: they have little or no effect on synaptic plasticity in the hippocampus (Snyder et al. 2016) and rather have been proposed to play a role in the amplification of signal transduction (Su and Hayashi 2003). Although it seems unlikely, we cannot completely exclude that sigma receptors contributed to remoxipride-mediated effects in our study.

## Conclusions

This study shows that the FN plays an important role in the acquisition of skilled reaching and goal-directed behavior and may also modulate motivation for this task. Furthermore, we show that D1R play a dominant role in this process, whereby receptor antagonism significantly impaired reaching skill acquisition and reduced task engagement. By contrast, D2R modulates consolidation. This indicates that a division of labor exists with regard to the role of FN DA receptors in the modulation of acquisition and stabilisation of skilled reaching competence. These findings advance our understanding of the role of the FN in reward-driven action and its dopaminergic contribution to skill acquisition and goal-directed behavior.

### Supplementary Information

Below is the link to the electronic supplementary material.

Supplementary file1 (PDF 140 KB)

## Data Availability

The datasets generated during and/or analysed during the current study are available from the corresponding author on reasonable request.
