# Supplementary material for: Dopamine receptors of the rodent fastigial nucleus support skilled reaching for goal-directed action
Source: Brain Struct Funct. 2023 Aug 24;229(3):609–37. doi: 10.1007/s00429-023-02685-0 (PMC10978667; doi:10.1007/s00429-023-02685-0)
Supplement: Supplementary file 1 — Supplementary file1 (PDF 140 KB) [file 429_2023_2685_MOESM1_ESM.pdf]

Supplementary Information

Article title: Dopamine receptors of the rodent fastigial nucleus support skilled reaching for goal-directed action

Journal name: Brain Structure and Function

Author names: V. M. Caragea, M. Méndez-Couz, D. Manahan-Vaughan

Affiliation and e-mail address of the corresponding author: D. Manahan-Vaughan, Department of Neurophysiology, Faculty of Medicine, Ruhr-University Bochum, Germany, denise.manahan-vaughan@rub.de

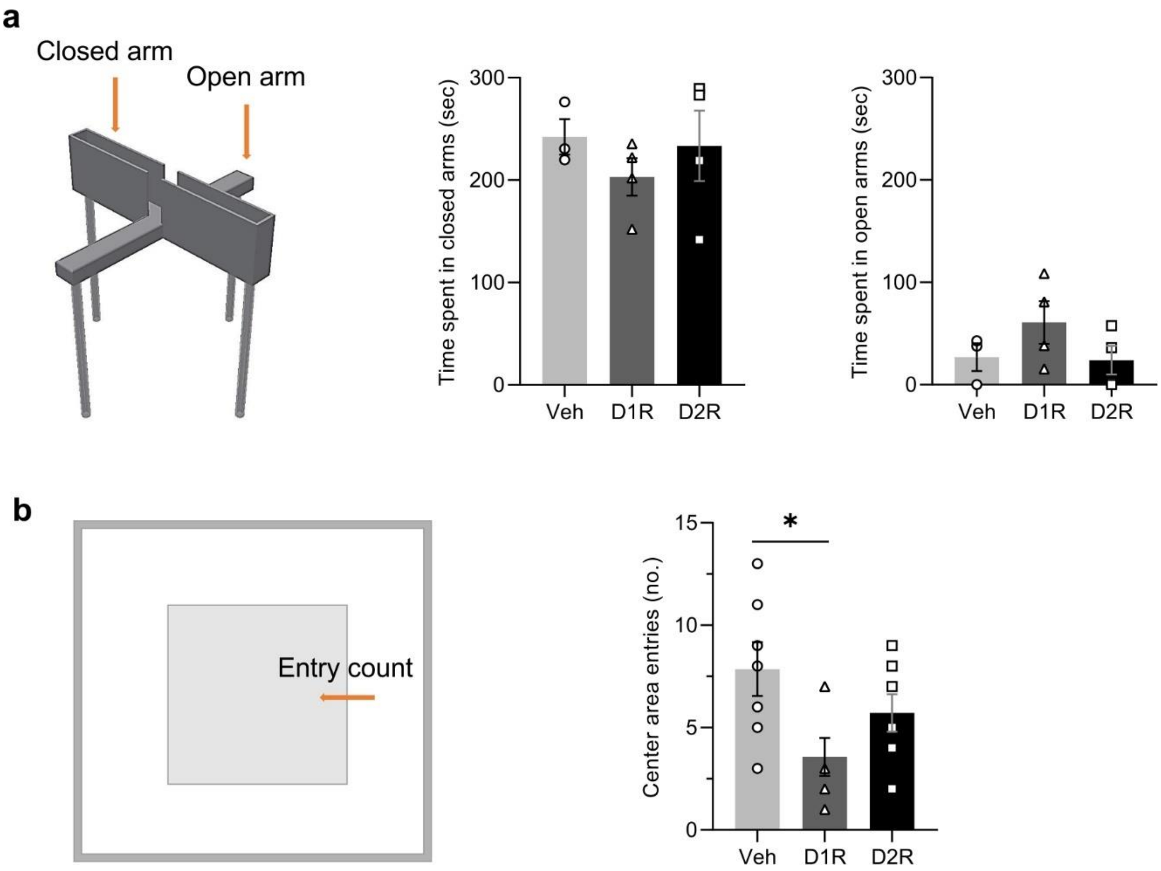

Figure S1. Motivation to explore was affected by D1R antagonist infused into the rat fastigial nucleus (FN)

a No differences were found in time spent in closed (middle panel) or open arms (right panel) of the elevated plus maze between treatments. Left panel illustrates the schema of the set-up used, where orange arrows indicate open and closed arms.

b D1R antagonist treated animals were less motivated to explore in the open field test. The left panel illustrates the open field arena used to measure center area entry counts (marked by orange arrow). The right panel shows a comparison of total center area entry counts between treatments, where the D1R antagonist group crossed this area less than the vehicle (*$p < 0.05$, $n = 7$, unpaired t-test).

For all panels: Veh = vehicle treated group; D1R = D1R antagonist treated group; D2R = D2R antagonist treated group. For more statistics, see Table S1.

Table S1. Statistical data for motivation-related experiments

**Task and measure**	**Veh**	**D1R**	**D2R**
Elevated plus maze	Mean (*SEM*), n=3	Mean (*SEM*), n=4	Mean (*SEM*), n=4
Time in closed arms	242.3 (*17.37*)	203.1 (*18.30*)	233.4 (*34.38*)
Time in open arms	26.83 (*13.50*)	60.76 (*20.91*)	23.92 (*14.00*)
Open field test	Mean (*SEM*), n=7	Mean (*SEM*), n=7	Mean (*SEM*), n=7
Center area entries	7.86 (*1.32*)	3.57 (*0.92*)	5.72 (*0.92*)

Abbreviations: *SEM* - standard error of the mean; *n* – number of animals used
